# Ramp-shaped neural tuning supports graded population-level representation of the object-to-scene continuum

**DOI:** 10.1038/s41598-022-21768-2

**Published:** 2022-10-27

**Authors:** Jeongho Park, Emilie Josephs, Talia Konkle

**Affiliations:** 1 Department of Psychology, Harvard University, Cambridge, USA; 2 Computer Science & Artificial Intelligence Lab, Massachusetts Institute of Technology, Cambridge, USA

**Keywords:** Perception, Object vision

## Abstract

We can easily perceive the spatial scale depicted in a picture, regardless of whether it is a small space (e.g., a close-up view of a chair) or a much larger space (e.g., an entire class room). How does the human visual system encode this continuous dimension? Here, we investigated the underlying neural coding of depicted spatial scale, by examining the voxel tuning and topographic organization of brain responses. We created naturalistic yet carefully-controlled stimuli by constructing virtual indoor environments, and rendered a series of snapshots to smoothly sample between a close-up view of the central object and far-scale view of the full environment (object-to-scene continuum). Human brain responses were measured to each position using functional magnetic resonance imaging. We did not find evidence for a smooth topographic mapping for the object-to-scene continuum on the cortex. Instead, we observed large swaths of cortex with opposing ramp-shaped profiles, with highest responses to one end of the object-to-scene continuum or the other, and a small region showing a weak tuning to intermediate scale views. However, when we considered the population code of the entire ventral occipito-temporal cortex, we found smooth and linear representation of the object-to-scene continuum. Our results together suggest that depicted spatial scale information is encoded parametrically in large-scale population codes across the entire ventral occipito-temporal cortex.

## Introduction

When we see a picture of an environment, we can easily perceive the spatial scale depicted in the image. Even in images that are the same 2-dimensional size, some may depict a very small space (e.g., a close-up view of a chair; typically referred to as “object” stimuli), whereas others may depict a much larger space (e.g., a view of an entire classroom; typically referred to as “scene” stimuli). Although objects and scenes have been typically treated as contrasting categories with a clear distinction^1–6^, this example illustrates that views of objects and views of scenes can be considered as opposite ends of a single dimension, namely of the spatial scale depicted in the current view^1^. The dimension of depicted spatial scale is continuous, with numerous intermediate-scale views between the two extremes (hereinafter, the “object-to-scene continuum”; c.f.^7^). Here, we examined the tuning and topographic organization of brain responses along the visual system to views varying along this continuum.

Foundational work has isolated and explored many scene-relevant factors, including global spatial frequency information or clutter, extended isolated contours, and other spatial distributions of rectilinearity and curvature^8–17^. It is now clear that many, if not all of these factors, jointly contribute to the response structure of scene-preferring regions, contributing largely shared variance across low-, mid-, and high-level features in the structure of brain responses^9^, (see^18,19^ for reviews). Related research on visual object representation indicates that many “high-level” tuning properties like category are also realized through tuning differences capturing more primitive visual featural distinctions^20–22^. Thus, here we assume that brain regions with systematic response variation to different “high-level” depicted spatial scales should not be interpreted as containing an explicit, abstract encoding of this dimension independent of all other dimensions, but instead arises through tuning to particular combinations of complex visual feature detectors^23^. Our approach is to tightly control the physical space that is visible in the view, while varying scene category and object content, thus allowing our design to explore whether any remaining systematic differences in image statistics related to the depicted scale evoke systematic responses along the ventral visual stream.

There are well-established brain regions that show selective preference for either end of the object-to-scene continuum (for reviews, see^24–27^). An object-preferring lateral occipital complex (LOC)^28^ is located on the lateral bank of the fusiform gyrus, and a scene-preferring parahippocampal place area (PPA)^5^ is located on the medial side of the inferior temporo-occipital cortex. Further, in recent work considering brain responses evoked by a particular level of intermediate scale views (“reachable-scale” views), we found regions that were activated more by images of reachable-scale environments (e.g., office desktops or kitchen counters) than either close-scale object views or navigable-scale scene views^29^. This work also showed that object- and scene-preferring regions both showed an intermediate level of activation to reachspace views (compared to object and scene views). Taken together, current research has demonstrated that different parts of the ventral visual stream have peak responses to views with different depicted spatial scales.

However, the research to date leaves open the question of how the full sweep of the object-to-scene continuum is encoded across visual cortex. One possibility is that depicted spatial scale preferences are smoothly mapped along the cortical surface, akin to retinotopic maps, where regions with tuning to adjacent parts of the visual field are also adjacent on the cortical sheet^30^. For example, the full object-to-scene continuum might be encoded with a contiguous bank of narrow Gaussian tuning functions, which show the highest activation to a particular depicted spatial scale, organized smoothly along the cortical surface. We will refer to this hypothesis as the “multi-channel model”^31^. The representation of orientation and spatial frequency in early visual cortex follows such a multi-channel encoding scheme, where the spatial arrangement of neurons with different preferred orientations form “orientation pinwheels”, reflecting a radial-shape preference map along the cortex^32–34^. Thus, one possibility for topographic organization of varying depicted spatial scale is a continuous map^35^, for example containing a continuous bank of response peaks to different points along the object-to-scene continuum, connecting the object-selective LOC, scene-selective PPA, and the more posterior ventral reachspace patch (vRSP) in between.

Another possibility is that depicted spatial scale drives opposite and balanced parametrically increasing (or decreasing) responses in different regions. That is, activity across spatial scale may be characterized in a given region-of-interest by maximum response to one end of the continuum, and a parametric, monotonic decrease in activity (linear or non-linear) toward the opposite end. For example, systematically increasing responses were found in the PPA and RSC as the perceived distance to objects (with a scene background) was rated from near to far^8^. Similarly, slight but systematically stronger overall responses were found in scene-selective regions as the depicted scale increased from the size of a walk-in closet to a large-scale arena^36^. Interestingly, they also found opposing response pattern in LOC, which showed *decreasing* responses as the depicted spatial scale increased. Under this scheme, an intriguing possibility is that information about the depicted spatial scale could be captured at a population level, in which neural sub-populations respond maximally to either extreme of the dimension, and depicted spatial scale information is encoded in the balance of these opposing responses^37^. This scheme would predict a more abrupt transition in tuning preferences along the cortical surface, as adjacent populations transition from near-preference to far-preference. However, this opponent coding model does not necessarily require or predict the existence of the intermediate-scale tuned regions^29^. More generally, there are other theoretically possible kinds of response profiles as depicted scale varies (e.g. a region with bimodal tuning, with the highest responses to both extremes). At stake with these different response schemes is how information about the depicted scale is reflected in a population code, with corresponding implications for how it can be read-out from the structure of brain responses.

**Figure 1:**
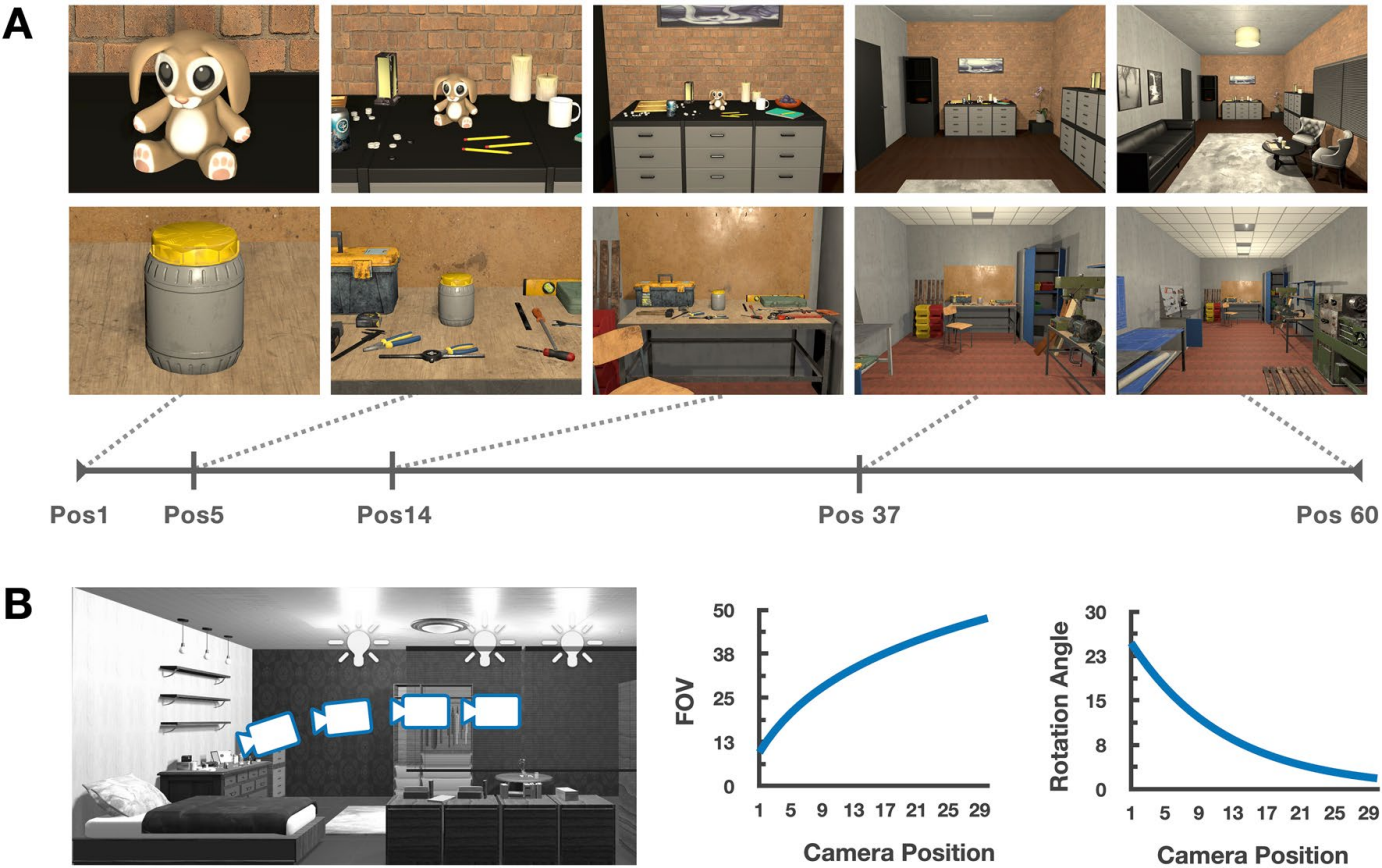
Stimuli. (**A**) Example images taken from two different environments (each row). Within each environment, we varied the the camera position along 15 log-scaled points, from a close-up object view (Position 1) up to a full-scene view (Position 60). Here, 5 positions are shown for illustration purposes. (**B**) Two camera parameters were systematically varied with the change of camera position. First, the field of view (FOV) was smallest at the closest view, then increased logarithmically as the camera moved toward the farthest view. Second, the rotation angle was downward at the closest view, then gradually adjusted upward, simulating a person looking at the central object from different positions in the room.

To explore these possibilities, we first built virtual 3-dimensional naturalistic indoor environments, sampling various scene categories with their corresponding objects and surface features. Critically, these environments all had identical physical dimensions, including a counter/surface with a central object at the back wall. Within each environment, we captured a smooth continuum of views by taking snapshots of the environment as if walking through the scene, with a camera position that moved through the environment and remained oriented towards the central object on the approach (see Fig. 1). As such, this ecological sequence of snapshots naturally reflects a number of changing properties that systematically vary along this continuum, from differences in low-level features to high-level affordances (e.g. spatial frequency content, texture gradients, object content, navigability). We measured human brain responses using functional magnetic resonance imaging, while participants viewed these naturalistic views of indoor environments. Our analytical approach was to (i) characterize the response properties of the ventral stream along the object-to-scene continuum, (ii) differentiate the underlying response profiles using a data-driven clustering method, and (iii) explore the nature of the population code using simulations.

## Results

### Cortical mapping of the object-to-scene continuum

Is there a large-scale topographic organization that smoothly maps the object-to-scene continuum (Fig. 1A)? To investigate this question, we showed participants snapshots of virtual indoor environments that were captured at various viewing distances from a central object. We created 20 different environments that had identical physical size and spatial dimensions. The environments included several semantic scene categories (e.g., kitchens, bedrooms, offices, etc), and each environment was populated with both small (e.g., tools) and big objects (e.g., cabinets) that are consistent with its category. Importantly, by the back wall of each environment, there was a “central object” on top of some desk-like structure (e.g., Fig. 1A).

The viewing distance for a given snapshot was defined as the distance from this central object to the position of the camera. This viewing distance was used as a proxy for the depicted spatial scale of a view, and was the key manipulated variable in our experiments. We varied the position of camera along 15 log-scaled points spanning from directly in front of the object to the back of the room (see Fig. 1B, left for a depiction of a sample views along this continuum). To ensure that the closest view was more object-focused, while the farthest scale view was more scene-focused, we had to make two further parallel adjustments to the camera parameters along this continuum. First, we adjusted the field of view of the camera, so that the closest view was a closer crop of the object, consistent with how object images are typically depicted to study object responses in the scanner; the field of view increased logarithmically with each step away from the object, to arrive at a more typical scene-focused view at the farthest position (Fig. 1B, middle). Second, we also adjusted the angle of the camera as a function of position (simulating a person with a fixed height, who must angle their head down on the approach to an object); the camera was angled downward to center the object at the closest distance, and the angle was gradually adjusted upward until it was parallel to the floor plane (Fig. 1B, right). See https://osf.io/hcmgk/ for all stimuli.

In the fMRI scanner, participants viewed images presented in a standard blocked design, while performing a one-back repetition detection task. A given block contained views from the same viewing distance (i.e., depicted spatial scale), sampled across different environments. Beta weights were extracted for each level of depicted spatial scale, for each voxel in each participant (Talairach space). Then, we computed the group-level betas by averaging betas across participants for each condition. Voxels used in subsequent analyses were selected based on the voxel-wise reliability within an anatomical mask^38^, which included occipito-temporal cortex (OTC), occipito-parietal cortex, and the corresponding medial part of the brain (see Supplemental Fig. 1A).

**Figure 2:**
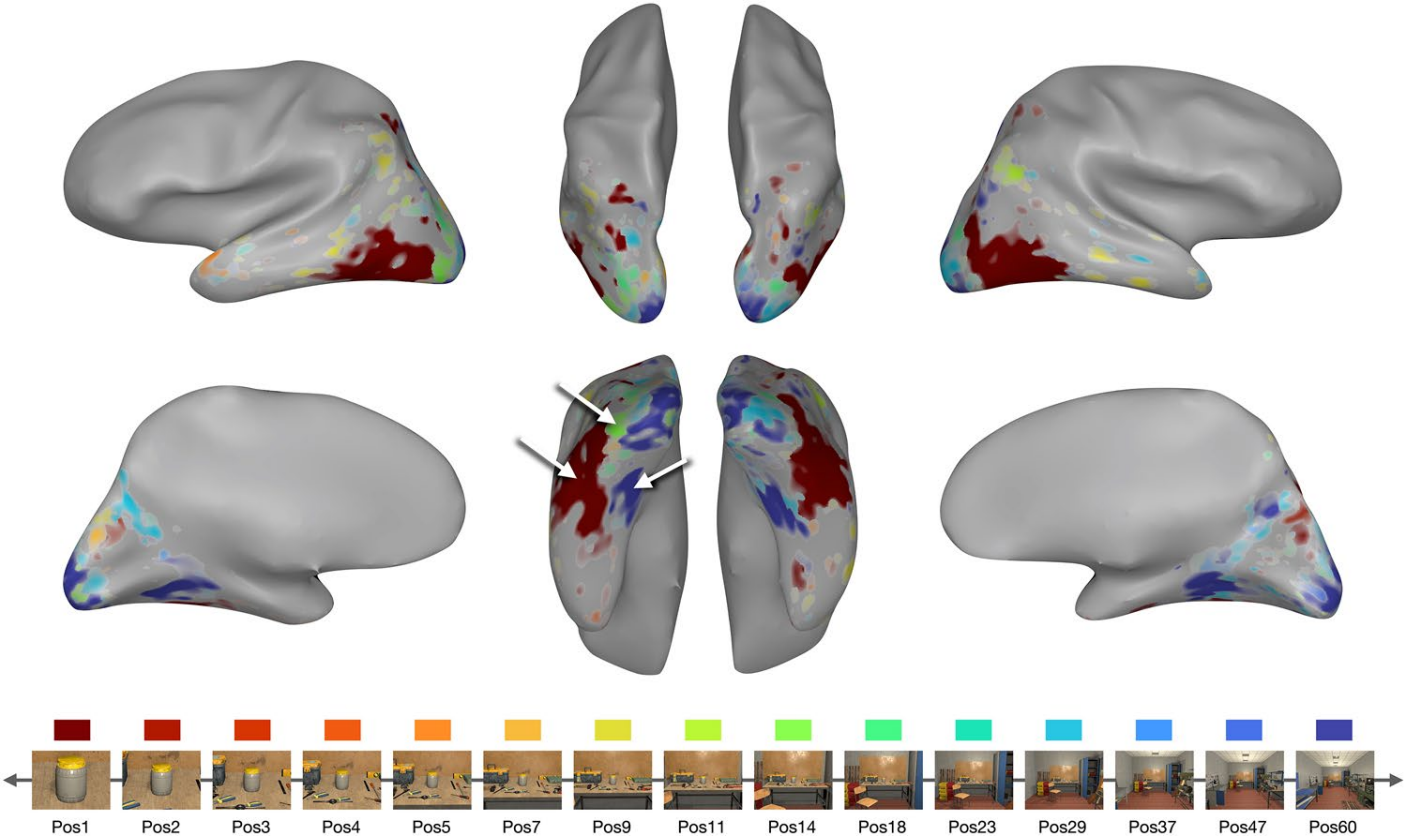
Preference map. Along the bottom, we show example images from 15 conditions depicting the object-to-scene continuum, and the corresponding color scheme for the plot above. The preference mapping result is visualized on an example subject brain, where each voxel is colored based on its most preferred condition (i.e., the highest activation), and the saturation is determined by the activation difference between the highest and the next highest condition. In the ventral occipto-temporal cortex (bottom middle), there is a large swath of cortex preferring the single-object views (dark red; white arrow) and another large territory preferring the far-scene views (dark blue; white arrow). There is also a small cluster preferring the intermediate-scale views (green; white arrow).

We first mapped the peak response preference of each voxel. Figure 2 shows these maps, in which each voxel was colored based on its preferred spatial scale (i.e., scale with the highest activation), where larger activation differences between the highest and the second highest condition are indicated with more saturated colors. The resulting preference map revealed extensive regions of cortex with a clear preference for either the closest object views or the farthest scene views. On the lateral surface, there was a large territory of cortex preferring the single-object view (dark red, Fig. 2); on the medial side, there was another large territory preferring the far-scene view (dark blue). Although somewhat smaller, there was also some portion of cortex preferring the intermediate scale (yellow to green colors, Fig. 2), in between the object-preferring and scene-preferring regions. While individual subject maps showed large individual differences (Supplemental Fig. 2A), the two clusters preferring the extreme close object view along the lateral cortex, and the extreme far scene-view along the medial cortex were consistently observed (Supplemental Fig. 2B). Overall, this preference map shows clear evidence for the preference of either extreme spatial scale, and some for the intermediate; we did not find clear evidence for a continuous mapping of the object-to-scene continuum across the cortical surface.

Additionally, we also examined the voxels’ preference by fitting a Gaussian to their response pattern over conditions. The preference mapping is sensitive to voxels that have a big activation difference between its most preferred condition compared to the next preferred one. However, there might be voxels that have smaller differences between conditions but in a systematic and meaningful way–e.g., a Gaussian shape with a wide standard deviation. To capture those, we estimated the peak of voxels by fitting a Gaussian function while varying its standard deviation. Broadly, we found a similar pattern to the preference map, which is that a majority of voxels prefer either extreme of the continuum and a few voxels prefer the intermediate scale (Supplementary Fig. 6).

### Data-driven response profile clustering

The preference mapping approach groups voxels based on their highest activation, implicitly assuming a voxel tuning shape with a single-peak. This approach might obscure other theoretically interesting tuning profiles. For example, some voxels might have preference for two different conditions (e.g., bimodal tuning), or some might have a parametric tuning across the conditions^36^. To test various response profiles of voxels without making a priori assumptions about them, we used a data-driven clustering approach, called response profile clustering (RPC)^39^. Specifically, we grouped voxels that have similar response profile using *k*-means clustering. As there were no constraints to group voxels into contiguous clusters, this approach also allows for the natural organizational structure to be revealed, based on differences in tuning along the object-to-scene continuum.

**Figure 3:**
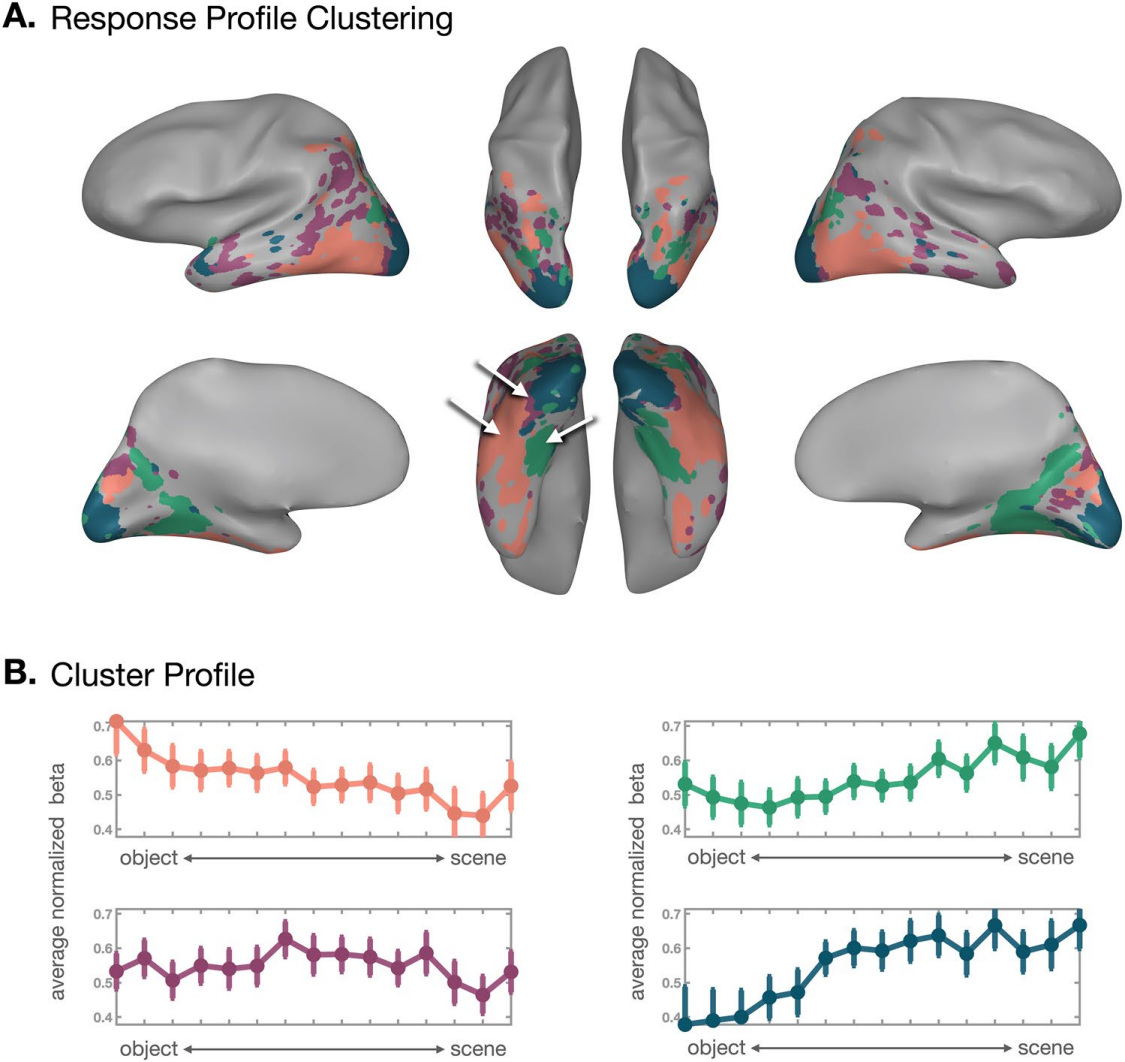
Response Profile Clustering. (**A**) The RPC results are visualized on an example subject brain (*k* = 4). Each voxel is colored based on its assigned cluster, and clusters with more similar response profiles have colors that are more similar in hue. The white arrows point to the three major clusters, and they are positioned in identical locations to Fig. 2 with respect to the brain. (**B**) The response profile of each cluster is plotted with the corresponding cluster color on the brain plot. The betas from each cluster are normalized to the grand mean and averaged across the voxels. The error bar represents +-1 standard deviation. The pink cluster shows the highest activation to single-object views and gradually less response toward the far-scene views. The green cluster shows the opposite pattern, where the highest activation was at the far-scene views. The purple cluster activation peaks for intermediate-scale views. The dark green cluster seems to have a similar response pattern to the green cluster, but it closely corresponds to independently defined early visual areas.

Here we report the response profile clustering solution with four groups of voxels (*k* = 4) and their corresponding response profiles. To select this number, we first computed a range of *k*-means solutions varying the number of clusters (*k*) from 2 to 20, and referenced the cluster center similarity and the Variance Ratio Criterion, which is a ratio between the within-cluster dispersion and the between-cluster dispersion (Supplemental Fig. 3A). The cluster similarity starts to plateau at *k* = 4 and above, suggesting that clusters are maximally different from each other at *k* = 3. Interestingly, however, the Variance Ratio Criterion showed a clear peak at *k* = 5. We chose to report *k* = 4 in the main figure, because we think the fourth cluster is worth noting as it corresponds well to the early visual areas manually delineated with independent data. Importantly, the major conclusions hold regardless of which *k* we pick. We report the rest of the cluster solutions in Supplementary Fig. 9. To visualize the clustering solution (*k* = 4), we created a cortical map, in which all voxels assigned to the same cluster were colored the same (Fig. 3A) and the corresponding cluster response profiles are plotted underneath (Fig. 3B, Supplemental Fig. 9).

Interestingly, three of the four data-driven clusters showed strong concordance with the zones which emerged from the preference mapping results. The first group of voxels (pink cluster) were mostly positioned at the lateral side of ventral occipital cortex. They formed a large-scale, spatially contiguous cluster, even though the clustering algorithm does not consider voxels’ anatomical locations or their spatial proximity to each other. This group of voxels showed the highest response to objects and gradually smaller response for scenes. The response change across the continuum was fairly smooth, resembling ramp-shaped tuning^40^.

In contrast, the second group of voxels (green cluster) covered a large swath of cortex at the medial side of ventral occipital cortex. This cluster showed the highest response to scenes and a graded response toward the object side of the continuum, with opposing ramp-shaped tuning. It is also noticeable that this cluster extended beyond the ventral region to the medial part where the retrosplenial cortex (RSC) is located, supporting this cluster’s preference for scenes. Results for additional category-selective regions are shown in Supplemental Fig. 5.

The third group of voxels (purple) is spatially positioned in between the first and second clusters, and showed the strongest response for intermediate-scale views. These three clusters were repeatedly found regardless of the number of clustering solutions (*k*) we choose, whereas other clusters were less robust and more variable depending on the *k*. The fourth group of voxels (dark green) near the occipital pole showed a similar pattern to the green cluster, and this cluster separates out from the green one only when k $\ge$ 4. However, it is worth noting that this cluster corresponds to an early visual cortex (V1–V3), which was separately defined with an independent data.

How robust is this clustering solution? We quantified robustness of the results in two ways. First, we assessed the reliability of the clustering solution across different subsets of stimuli. Given *k*, the clustering was performed separately for two subsets of data which were divided by the stimuli (e.g., environment set A vs. environment set B). Then, the agreement of clustering solutions was quantified using Dice coefficient and the *d*-prime (see Methods). This analysis showed consistent clustering results across different environments (Dice coefficient = 0.71, *d’* = 1.2; Supplemental Fig. 3C), demonstrating that the observed solution likely reflects some common features across environments (e.g., spatial scale or spatial layout) rather than environment-specific features (e.g., particular objects or textures). Second, we assessed the stability of the clustering solutions across participants. For each iteration (50 iterations), the participants were randomly split into two groups, and we measured how well one group predicts the other (see Methods). The results showed decreasing Dice coefficient as the number of cluster increases (0.26–0.76, mean = 0.49, s.d. = 0.1), but relatively stable *d’* scores across the whole range of *k*s (0.86–0.98, mean = 0.88, s.d. = 0.03; Supplemental Fig. 3B). While these two measures yield different patterns in terms of the “best” clustering solution and the correspondence between solutions as a function of *k*, the critical response profiles we emphasize (i.e., major clusters with either increasing or decreasing responses along the continuum) remain consistent across all clustering solutions.

Altogether, we did not find a smooth topographic mapping of the object-to-scene continuum in the peak responses along the cortical surface, considering both our preference mapping analysis and the more nuanced response profile clustering (RPC). Instead, these analyses revealed clear evidence for distinct swaths of cortical territory with opposing ramp-shaped tuning, with peak responses at either extreme of the object-to-scene continuum, and smaller clusters of voxels intermediate in anatomical position with intermediate tuning preferences. Given this kind of structure in the univariate response profiles of these voxels, we next examined the impact of such tunings on the representation of the object-to-scene continuum at a *population level*–that is, how are the distinctions between depicted spatial scale realized in the distributed population responses that span these differently-tuned clusters.

### Population-level response structure

**Figure 4:**
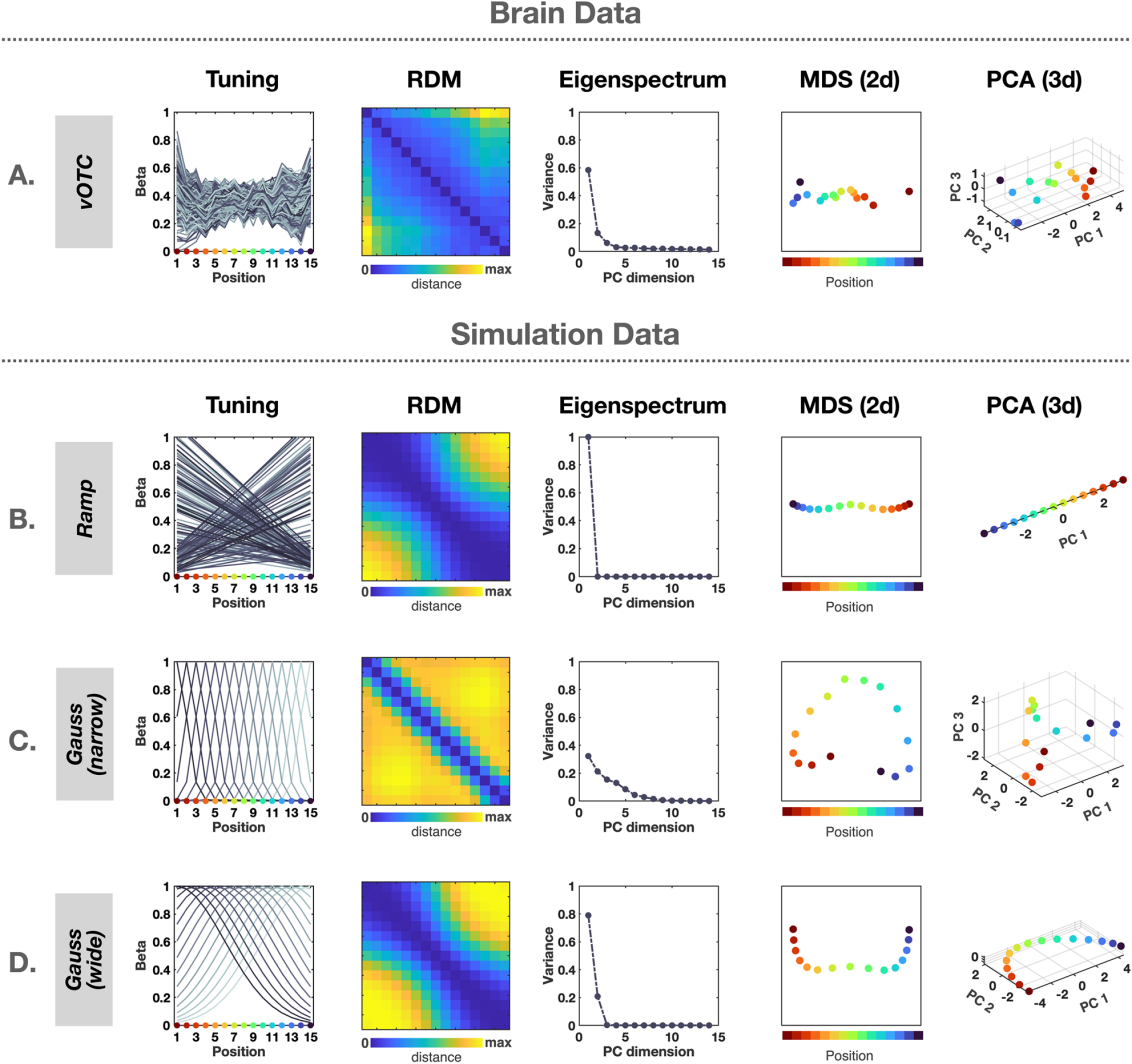
Neural tuning and representational geometry analysis. (**A**) Population of responses in the brain data. We show normalized voxel tunings over the object-to-scene continuum (Tuning), pairwise representational distances between conditions (RDM), how much variance is explained by each principal component (eigenspectrum), and visualization of representational geometry in the population (MDS (2d) and PCA (3d)). (**B**–**D**) Simulation idealized voxel tunings and representational geometry. The ramp-shaped tuning (**B**) shows one-dimension feature space with a perfectly linear trajectory, whereas the narrow Gaussian tuning (sigma = 1, **C**) shows much higher feature dimension with a cardioid-like trajectory. However, the wide Gaussian tuning (sigma = 10, **D**) showed similar representational geometry to the ramp-shaped tuning.

To investigate how depicted spatial scale is encoded in the population of voxels, we conducted several analyses to characterize and visualize the representational geometry^41^, depicted in Fig. 4A. While we focused on the ventral occipital temporal cortex (vOTC) here, including lateral OTC voxels did not make any qualitative changes in results, presumably because of similar voxel tuning profiles in those sectors. First, a representational dissimilarity matrix (RDM) was generated by computing the correlation between multi-voxel patterns of each condition pair across vOTC voxels (Fig. 4A). There was a smooth pattern along the diagonal in the RDM, indicating that images with similar spatial scales also have more similar neural patterns across vOTC voxels.

Next, inspired by analyses in Stringer and colleagues^42^, we conducted a principal component analysis (PCA) over the condition $\times$ voxel matrix, which gives a sense of the dimensionality of the population code over these conditions. The eigenspectrum—that is, the proportion of explained variance by each principal component—is shown in Fig. 4A. In the population of vOTC voxels, the first PC explained 58.5% of variance, followed by the second PC explaining 13.3% of variance, suggesting a relatively low-dimensional representation through the population in response to the continuum of objects and scenes. To visualize this geometry, we employed both a 2D multidimensional scaling (MDS) and in 3D principal component (PC) space (Fig. 4A). In both cases, a smooth pattern along spatial scale was clearly shown; in this population code, these object-to-scene conditions trace out a surprisingly linear trajectory along the major axis of variation. These analyses indicate that the depicted spatial scale of a view is represented systematically and parametrically in this larger population level code.

To further explore the relationship between voxel tuning and the resulting structure and dimensionality of the population-level representational geometry, we performed simulations with idealized voxel tuning profiles. First, we modeled pure ramp-shaped tuning, with two pools of simulated voxels tuned to each extreme end of the object-to-scene continuum (Fig. 4B, see methods). Second, we modeled narrow Gaussian tuning along the continuum, with different units tuned to different scales along the continuum (Fig. 4C).

The resulting representational geometry of these simulated voxel populations revealed clear differences between the two tuning motifs. For the ramp-shaped tuning, we observed an RDM with a broad diagonal and the first PC explaining 100% of the variance, with conditions mapping out a perfectly linear trajectory in the population code. In contrast, the narrow Gaussian tuning showed an RDM with a sharper and clear diagonal, a higher dimensional code (i.e., more curved eigenspectrum), with a curved (cardioid-like) trajectory in the population code. Qualitatively, the ramp-shaped tuning shows more similar signatures to the brain data than the narrow Gaussian tuning encoding scheme. To quantify the relationship between the neural data and the simulated models, we computed the similarity between each subject’s neural RDMs and each of the simulated models using Spearman correlation. The correlation between the neural RDM and the ramp-shaped RDM (mean = 0.64, se = 0.06) was significantly higher than the correlation between the neural RDM and the narrow Gaussian RDM (mean = 0.5, se = 0.04; *t*(11) = 7.02, *p* < 0.01).

However, when we increased the width of Gaussian (i.e., broader tuning), the representation geometry becomes increasingly similar to the ramp-shaped tuning simulation. The correlation between the neural RDM and the wide Gaussian RDM (mean = 0.64, se = 0.06) was significantly different from the correlation between the neural RDM and the narrow Gaussian RDM (*t*(11) = $-$5.36, *p* < 0.01), but it was not significantly different from that of the ramp-shaped RDM (*t*(11) = 0.52, *p* = 0.62). This is because wide Gaussians with peak tuning closer to the extreme ends of the continuum start to resemble the opponent ramp tuning functions, as shown in the RDM visualization (Fig. 4D). Thus, in practice, it is difficult to distinguish these two coding schemes, even though they have very different theoretical implications^31,37^. However, one apparent difference between them is that with the wide-Gaussian model, the eigenspectrum does not collapse to 1 dimension, as there are at least some voxels with a preference at the middle of the continuum which cannot be modeled by combinations of two ramp shaped functions. This qualitative pattern in the eigenspectrum is actually slightly more similar to the vOTC voxels, and is perhaps consistent with the fact that while most of the voxels showed ramp-shaped tuning with peaks at the extreme, we also found a small cluster of voxels with peak tuning at an intermediate scale.

Thus, our exploratory simulations overall reveal that monotonically increasing/decreasing tuning functions (either from the ramp-shaped or wide Gaussian) are important for a parametric representation of depicted spatial scale in the population code. By having these two pools of voxels that are tuned to the opposite extremes, the object-to-scene continuum is clearly represented as linear trajectory through this large-scale population of voxels, parametrically as a function of depicted spatial scale. These simulations also provide evidence against a coding scheme in which different neural sub-populations are tightly tuned with different peaks along this depicted scale dimension; however, they also leave open the possibility that there may be wider tuning curves, or some other encoding scheme, as none of the simulations qualitatively capture the smooth curve of the eigenspectrum and the blockier similarity structure evident in the brain data RDM.

## Discussion

In this study, we investigated the structure of visual brain responses to ecological variation in the depicted spatial scale of a visual scene. Using rich virtual environments with controlled spatial layouts, we tested a range of depicted spatial scales from a close-up object view to a full scene view, densely sampling views in between. Considering voxel tuning, we found evidence for large swaths of cortex with opposing ramp-shaped tunings, with a smaller region with weak intermediate scale tuning. Considering the corresponding implications for the structure of multi-voxel patterns across this entire cortex, we observed that object-to-scene continuum was represented smoothly in the population code. Our simulations confirmed that in order to induce such representational geometry, it is crucial for individual voxels to show monotonic increasing or decreasing voxel tunings, which are the tuning profile we observed in the response profile clustering. Altogether, our results show that the depicted spatial scale of a view is represented parametrically in a large population of voxels, rather than with a smooth continuum of response preferences along the cortical surface.

### Independent versus competitive populations

Here we posit two populations with opposing ramp-shaped responses across the object-to-scene continuum. What features drive responses in each of these populations, and how do they relate to each other? One possible account is that each ramp-shaped population is coding complementary aspects of a visual environment in parallel, and *independently*^23^. This account is compatible with previous works^15,43^, which argued that the PPA represents the spatial boundaries of a scene (e.g., walls and floors) and the LOC represents the content of the scene (e.g., textures, materials, or objects). In our data, the ramp-shaped tunings were found in two distinctive clusters in the vOTC beyond the PPA and LOC, which suggests that those responses reflect not only category-specific features that functional ROIs are sensitive to, but more general visual features. In fact, the data pattern is consistent with previous findings showing sharp dissociation along the mid-fusiform sulcus (MFS)^44^, where there are clear lateral to medial transitions in anatomical features such as cytoarchitecture^45^ or white-matter connectivity^46^, as well as functional responses, such as eccentricity bias^47^, animacy^48^, or real-world object size^49^. Based on these, it was argued that visual information is sorted and mapped onto different sides of the MFS, as a solution to the problem of organizing high-dimensional information along the 2D cortical sheet^44^. Under this account, the ramp-shaped responses across depicted spatial scale in these regions are a natural consequence of the way different combinations of visual features naturally co-vary with ecological changes in view from object-focused to scene-focused views.

An alternate possibility is that the responses of these two populations are not independent, but are competitive and inter-related. For example, there might be connectivity that ensures that when one population is activated high another population is suppressed. In fact, there is some evidence for such competitive relationship^50^. When the LO was disrupted using transcranial magnetic stimulation (TMS), the response in the PPA increased for scene stimuli, suggesting inhibitory connections between them. Prior work has shown computational advantages to coupling two opposing ramp-shaped tuning functions^51^. For example, it allows for simple and efficient read-out by providing linearly separable representations^44,52^, in a way that is robust to baseline activation changes (e.g., from adaptation^53^), and enables fine-grained discrimination, as an exact position can be computed as a balance of two populations.

Although we cannot tease apart these two accounts here, it may be possible to test them by independently manipulating different aspects of images. For example, we could create identical environments but without much object content while leaving the spatial structure intact. The first account predicts that responses in only one of populations will be affected (e.g., object-processing systems), whereas the second account predicts that both populations will be affected due to their connections. Thus, the present work clarifies the need for further research aimed at characterizing these two regions jointly, delineating the relationship between the underlying feature tuning and potential competitive interaction between these two populations with opposing ramp-shaped tuning.

### Connections with related work

To what extent our results are consistent with previous studies that asked similar questions? First, we found a small cluster of voxels showing preference for the intermediate spatial scale, in addition to the large clusters with ramp-shaped tuning. The strength of preference was relatively weak, but it was consistently observed across several analyses. This intermediate-preferring cluster might be consistent with reachspace-preferring regions^29^. However, the anatomical locations of these reachspace-preferring regions were not clearly mapped onto the intermediate-preferring cluster in the current study. It is partly because many voxels in the reachspace-preferring region did not survive the voxel selection threshold in our data. Thus, we cannot draw a firm conclusion here (see Supplemental Fig. 4 for details).

Second, while our data could not clearly disambiguate between the ramp-shaped tuning and wide Gaussian tuning, it clearly showed that there is not a narrow Gaussian tuning along the dimension of depicted spatial scale. This pattern of data may seem at odds with the claims of Peer and colleagues^54^. Do our results contradict their findings? On the surface, the two studies may seem similar, but there are some fundamental differences. First, there were no visual stimuli presented, except the written words of objects for the task. Thus, the representation of spatial scale captured in the previous study could be much more abstract or modality-independent compared to the representation of visually depicted spatial scale in the current study. Second, the task in our study was passive viewing of visual scenes, whereas the task in the previous study^54^ was judgments of the distance between objects. Finally, the range of spatial scales used in the previous study is much larger than our study, e.g. their closest spatial scale level was indicated by the word ‘room’, followed by ‘building’, while in our study, the farthest condition was a view of an entire room. These inherent differences between the two studies thus help situate the different findings between the present work and that of Peer and colleagues^54^.

### Depicted spatial scale and meaningfully co-varying dimensions

In this study, we allowed low, mid, and high-level properties to naturally co-vary as they arise along an ecologically sampled continuum of object-focused to scene-focused views. This covariance raises a natural reductionist line of inquiry–are these responses really *about* depicted scale, or are they better explained by tuning along other visual properties?

For example, does the present organization by depicted spatial scale simply reflect the object size organization? Object size is a known factor in cortical organization, with lateral ventral cortex responding more to small objects and medial responding more to large objects^49^. The typical visual size of objects is tied to the spatial scale of the view: at a close scale, only small objects fit in the view, and at the far scale, large objects fill and define the space. Thus, it is likely that responses across this cortex that vary by object size and depicted spatial scale are partially related: the visual sizes of the objects constitute some of the basic visual statistics that co-vary with depicted scale. However, the organization by depicted spatial scale that we describe here is not likely to be entirely reducible to object size. First, while scene preferring regions do respond to single objects (especially large and space-defining ones)^8,55^, we know that a large component of their response is accounted for by the global scene elements such as walls and floors^5,15,16^. Thus, previous work strongly suggests that responses in medial ventral regions are not reducible to object size responses. Second, recent work suggests that cortical regions that are responsive to intermediate-scale views respond more to multi-object arrays than individual objects^29^. Thus, we see object size as one factor among many that vary as a function of the spatial scale of a depicted view in everyday scenes.

Indeed, as is increasingly evident in the study of object category representation, the tuning of this part of the ventral visual stream is largely related to mid-level image statistical properties (rather than abstract high-level categories or properties^20^). Similar tuning at mid-level image statistical properties are likely the underlying causes of the differential tuning by depicted spatial scale of the environment. For example, an analysis of the image statistics present across different environments indicates that there is increasing high spatial frequency content with increasing depicted scale (F(14, 285) = 3.54, *p* < 0.01; Supplementary Fig. 8). Previous early computational models of natural image statistics showed that there are different distributions of spatial frequency content across a scene (e.g. differences in the top and bottom part of the image), which can be used to decode the spatial scale of the depicted environment^56^. In this framework, the distribution of spatial frequency information in a scene image is not a confounding factor with spatial scale, unrelated to the dimension of interest, but rather one of the early visual features that the visual system likely leverages to support the high-level property inference. More broadly, such image-computable models that enable read out of ’high-level’ properties of the scene make important strides towards our ability to discover the nature of the complex feature tuning underlying these visual brain responses^57,58^.

### Conclusion

In summary, we examined how the human visual cortex responds to the object-to-scene continuum. We did not find strong evidence for a topographic map on the cortex, where adjacent parts of the cortex systematically map adjacent parts of the object-to-scene continuum. Instead, we found two opposite ramp-shaped tunings, and a smooth representation of the object-to-scene continuum emerged only in the structure of the larger population code. One important thing to note is that the object-to-scene continuum tested in our study is the pictorial description of spatial scale, which was achieved by adjusting the field of view as well as viewing distance. However, in natural viewing conditions, the vergence of the eyes on an object changes the human field of view much less dramatically than implied here, and we have access to the full-field of view without an image border. Thus, one promising future direction to take this research in an even more ecological direction would be to test the perception of spatial scale in a full-field viewing setting, allowing far-peripheral vision to be engaged in scene processing, alongside central vision as tested here.

## Methods

### Participants

Twelve participants (5 females, age: 20–38 years) with normal or corrected-to-normal vision were recruited from the Harvard University community. All participants gave informed consent and were financially compensated. The experiment was performed in accordance with relevant guidelines and regulations, and all procedures were approved by the Harvard University Human Subjects Institutional Review Board.

### Stimuli

Computer generated (CGI) environments were generated using the Unity video game engine (Unity Technologies, Version 2017.3.0). We constructed twenty indoor environments, reflecting a variety of semantic categories (e.g., kitchens, bedrooms, laboratories, cafeterias, etc.). All rooms had the same physical dimensions (4 width $\times$ 3 height $\times$ 6 depth arbitrary units. in Unity), with an extended horizontal surface along the back wall, containing a centrally-positioned object. Each environment was additionally populated with the kinds of objects typically encountered in those locations, creating naturalistic CGI environments.

Images spanning a continuum of distances from the central object were captured from each environment (1024 $\times$ 768 pixels, approximately 20 visual angles wide), ranging from a close-up view of the object to a far-scale view, which included the whole room. Images were generated by systematically varying the location of the camera (hereafter “Position”) along 15 log-scaled points arrayed from the “front” to the “back” of the room (i.e., from right in front of the central object to across the room from it, Fig. 1A). Close-up views were captured with a smaller camera field of view (FOV), so that only the central object appeared in the frame, and the FOV increased logarithmically with each step away from the object. The camera angle was parallel to the floor plane for far-scale views, and was gradually adjusted downward for closer positions, so that the central object was always at the center of the image Fig. 1B). These camera parameters were used for all 20 environments, yielding 300 unique stimuli (20 environments $\times$ 15 positions). See https://osf.io/hcmgk/ for all stimuli.

### Experimental design

The main experiment consisted of 10 runs. Each run was 6.2 min in duration (186 TRs), and used a standard blocked design, with 15 conditions presented twice each run. We chose the block design to detect activation that is common across the stimuli at each spatial scale (e.g., structural regularities of environments at a particular spatial scale^56^). Each condition block was 6s, in which five images from one condition were presented (e.g., 5 different environments viewed the same position), and was always followed by a 6s fixation period. Within a condition block, each image was presented for 800 ms, followed by a 300 ms blank screen. The presentation order of blocks in each run was pseudo-randomized as follows. Fifteen conditions within an epoch were randomized twice independently and concatenated with a constraint that the same condition cannot appear in two successive blocks. The CGI environments were randomly divided into two sets (Environment Set A and B) and used for odd and even runs. These stimuli sets were kept the same within each participant. Participants performed a red frame detection in which they pressed a button whenever there was a red frame surrounding the stimulus. The red frame appeared once in a block, in a random position among 5 images. Participants were instructed to pay attention to both spatial layout and objects.

Two functional localizer runs were performed independent of the main experimental runs, each 6.9 min (208 TRs). In each run, participants saw blocks of faces, objects, scenes, and reachspaces images^29^, while performing one-back repetition detection task. In each condition block (8 sec), seven unique images were selected and one of those images was randomly chosen and repeated twice in a row. Participants were instructed to press a button when they saw the repeated image. In each trial, an image was presented for 800 ms, followed by a 200 ms blank screen. Ten blocks per condition were acquired within a run. The presentation order of blocks in each run was randomized within each epoch. One epoch consisted of one block from each of four conditions and one fixation block (8 sec). This procedure was repeated ten times and the block orders were concatenated across the epochs.

Finally, two additional retinotopy runs were performed, each 5.4 min (162 TR). This protocol consisted of 4 conditions: horizontal bands ($\sim 22^{\circ } \times 1.7^{\circ }$), vertical bands ($\sim 1.7^{\circ } \times 22^{\circ }$), central stimulation (radius $\sim 1.2^{\circ }$ to $2.4^{\circ }$), and peripheral stimulation (radius $\sim 9.3^{\circ }$ to the edges of the screen). The horizontal and vertical bands showed checkerboards which cycled between states of black-and-white, white-and-black, and randomly colored at 6hz. The central and peripheral rings showed photograph fragments which cycled between patterns of object ensembles (e.g., buttons, beads) and scene fragments^12,59^. Each run consisted of 5 blocks per condition (12 sec block), with five 12 sec fixation blocks interleaved throughout the experiment. An additional 12 sec fixation block was added at the beginning and the end of the run. Participants were asked to maintain fixation and press a button when the fixation dot turned red, which happened at a random time once per block.

The stimuli presentation and the experiment program were produced and controlled by MATLAB and Psychophysics Toolbox^60,61^.

### fMRI data acquisition

All neuroimaging data were collected at the Harvard Center for Brain Sciences using a 32-channel phased-array head coil with a 3T Siemens Prisma fMRI Scanner. High-resolution T1-weighted anatomical scans were acquired using a 3D MPRAGE protocol (176 sagittal slices; FOV = 256 mm; 1x1x1 mm voxel resolution; gap thickness = 0 mm; TR = 2530 ms; TE = 1.69 ms; flip angle = 7$^{\circ }$). Blood oxygenation level-dependent (BOLD) contrast functional scans were obtained using a gradient echo-planar T2* sequence (84 oblique axial slices acquired at a 25$^{\circ }$ angle off of the anterior commissure-posterior commissure line; FOV = 204 mm; 1.5 $\times$ 1.5 $\times$ 1.5 mm voxel resolution; gap thickness = 0 mm; TR = 2000 ms; TE = 30 ms, flip angle = 80$^{\circ }$, multi-band acceleration factor = 3).

### fMRI data analysis and preprocessing

The fMRI data were analyzed with BrainVoyager 21.2.0 software (Brain Innovation) with custom Matlab scripting. Preprocessing included slice-time correction, linear trend removal, 3D motion correction, temporal high-pass filtering, and spatial smoothing (4mm FWHM kernel). The data were first aligned to the AC-PC axis, then transformed into the standardized Talairach space (TAL). Three-dimensional models of each participant’s cortical surface were generated from the high-resolution T1-weighted anatomical scan using the default segmentation procedures in FreeSurfer. For visualizing activations on inflated brains, the segmented surfaces were imported back into BrainVoyager and inflated using the BrainVoyager surface module. Gray matter masks were defined in the volume based on the Freesurfer cortex segmentation.

A random-effect group general linear model (GLM) was fit using BrainVoyager. The design matrix included regressors for each condition block (Position 1–15) and 6 motion parameters as nuisance regressors. The condition regressors were constructed based on boxcar functions for each condition, convolved with a canonical hemodynamic response function (HRF), and were used to fit voxel-wise time course data with z-normalization and correction for serial correlations. The extracted beta weights from this group GLM were averaged across participants for each voxel, and then taken as the primary measure of interest for all subsequent analyses. The group data was displayed on a selected participant’s cortical surface.

### Regions of interest (ROIs)

We identified five ROIs separately in each hemisphere in each participant, using condition contrasts implemented subject-specific general linear models. Three scene-selective areas were defined using Scenes—Objects (*p* < .0001) contrast. Specifically, the parahippocampal place area (PPA) was defined by locating the cluster between posterior parahippocampal gyrus and lingual gyrus, the retrosplenial cortex (RSC) was defined by locating the cluster near the posterior cingulate cortex, and the occipital place area (OPA) was defined by locating the cluster near transverse occipital sulcus. The lateral occipital complex (LOC) was defined using Objects—Scenes (*p* < .0001) contrast. The fusiform face area (FFA) was defined using Faces—Objects (*p* < .0001) contrast. Finally, the early visual areas (EVA; V1–V3) were defined manually on inflated brain, based on the contrast of Horizontal–Vertical meridians from the retinotopy runs.

### Voxel selection

The preference mapping and response profile clustering analyses were performed on voxels selected using the anatomical mask and the reliability-based voxel selection method^38^. First, we manually drew a mask in BrainVoyager (21.2.0) encompassing occipito-temporal cortex, occipito-parietal cortex, and the corresponding medial part of the brain. Within this mask (17,684 voxels), we calculated split-half reliability for each voxel by correlating the betas from odd and even runs of the main experimental runs. Based on the resulting correlation map, we chose *r* = 0.3 as the cutoff and selected voxels with higher reliability (6370 voxels, about 36% survived; Supplemental Fig. 1). This voxel selection procedure was performed on a group-level in the Talairach space.

### Preference mapping

To examine whether there is a topographic mapping of the object-to-scene continuum, we calculated a group-level preference map. First, responses to each of 15 Positions were extracted in each voxel from single-subject GLMs, then averaged over subjects. For each voxel, a condition showing the highest group-average response was identified as the preferred condition. The degree of preference was computed by taking the response differences between the most preferred condition and the next-most-preferred condition. To visualize these response topographies, we colored each voxel with a color hue corresponding to the preferred condition, with a color intensity reflecting the degree of preference. This preference map was projected onto the cortical surface of a sample participant. Similar preference mapping procedures have been used in the studies examining a large-scale cortex organization^20,29,62–64^.

### Response profile clustering

We used a data-driven neural clustering method to see whether there are clusters of voxels responding similarly over the conditions. Using MATLAB’s implementation of the *k*-means algorithm with the correlation distance, voxels were grouped based on their response profile similarity (10 replicates, 500 max iterations). To measure the similarity, the response profiles for all voxels were first transformed to have zero-mean and unit length, and then correlation was used as the distance metric. We chose to use the correlation in order to compare relative response magnitudes across the conditions, rather than differences in overall response magnitudes, which may be sensitive to a voxel’s anatomical location and signal measurement related reasons (e.g., proximity to head coils). The output of this analysis was a clustering assignment for each voxel, with a cluster response profile, which reflects the average normalized profile for all voxels included in the cluster. To visualize the clustering solution, we created cortical maps in which all voxels assigned to the same cluster were colored the same. For visualization purposes, we chose these colors in a way that clusters with more similar response profiles were more similar in hue. To do so, we submitted the cluster response profiles to a multidimensional scaling algorithm using a correlation distance measure, placing similar cluster centroids nearby in a three-dimensional space. The 3D coordinates of each point were re-scaled to be within [0 1], and then used as the Red–Green–Blue color channels for the cluster color.

To determine the number of cluster solutions, we computed a range of *k*-means solutions varying the number of clusters (*k*) from 2 to 20. To choose the final *k* to report, we referenced the cluster center similarity and the Variance Ratio Criterion (Calinski Harabasz score; Supplemental Fig. 3A). The cluster similarity measures how similar the cluster centroids are to one another on average, whereas the Variance Ratio Criterion measures the ratio between the within-cluster dispersion and the between-cluster dispersion.

For assessing the stability of the clustering solution, we conducted two additional analyses. First, we assessed the reliability of the clustering solution across different subsets of stimuli (Supplemental Fig. 3C). The data were split into two based on the stimuli (Environment Set A vs. Environment Set B), and the identical clustering algorithm was run separately for each data set. Then, we quantified how well those two clustering solutions converged, using two separate measures based on a signal detection method: Dice coefficient and D-prime.

For this analysis, we created a matrix (voxels $\times$ voxels), in which the value is 1 if the voxels were assigned to the same cluster and 0 if the voxels were assigned to different clusters, for each solution. Then, Hit rate was calculated as the percentage of voxel pairs that were assigned to the same cluster in one solution that were also assigned to the same cluster in another solution; and False Alarm (FA) rate was calculated as the percentage of voxel pairs that were not assigned to the same cluster in one solution but were assigned to the same cluster in another solution. Given this, the Dice coefficient was computed as [2*Hit/(2*Hit + FA + Miss)], accounting for both false positives and negatives. The D-prime was calculated as z(Hit) $-$ z(FA), reflecting the detection sensitivity against the false positives.

Further, we examined the response profile of clusters using a cross-validation method. The clustering solution was first obtained from one subset of data. Then, using this cluster assignment, we extracted betas from the remaining data. We repeated the same procedure the other way around (Supplemental Fig. 3C).

Second, we assessed the stability of the clustering solutions across participants (Supplemental Fig. 3B). For each iteration, the participants were randomly split into two groups, and how well one group predicted the other was computed using the Dice coefficient and D-prime, as described above. These metrics were measured for each of the solutions *k* = 2 to 20. Then, the entire procedure was repeated over 50 iterations, and we computed the average score over iterations for each clustering solution *k*.

### Population-level analyses

This analysis examined how the object-to-scene continuum is represented as the distributed patterns across voxels in the ventral occipitotemporal cortex beyond early visual cortex. We selected a set of voxels that were located within an anatomical mask of ventral occipitotemporal cortex (vOTC) and the voxel-wise reliability is higher than 0.3, while excluding the early visual cortex (V1–V3). Then, we computed the distance between each condition pairs by taking 1-Pearson correlation value, which resulted in a 15 $\times$ 15 representational dissimilarity matrix (RDM). The pairwise distance relationship between conditions was also visualized using a 2-dimensional classic multidimensional scaling (MDS) function implemented in MATLAB R2020B.

To further explore the geometry of the neural code, we performed a dimension reduction technique, principal component analysis (PCA), over the selected voxels. The PCA function was implemented in MATLAB R2020B. Then, we made an eigenspectrum plot which shows the proportion of variance explained by individual principal components. Finally, we visualized how each condition is represented in 3-dimensional PC space.

### Hypothetical voxel tuning simulation

To better understand the relationship between individual voxel tunings and their population-level representation, we ran simulations comparing two coding models with idealized voxel tunings: (1) multi-channel coding with Gaussian tuning, and (2) two-opponent coding with ramp-shaped tuning. For the multi-channel model, we generated simulated activations of 150 voxels based on a Gaussian distribution. Note that we varied the number of voxels and found that this does not change the results. The $\mu$ of Gaussian distribution was randomly chosen between 1 and 15 (i.e., corresponding to each stimulus condition), from the discrete uniform distribution. We chose to use discrete sampling, because the main purpose of the simulation was to emulate the activation pattern we measured (with discrete conditions), rather than directly modeling the tuning over spatial scale (continuous). To examine how the representational geometry changes depending on the width of Gaussian tuning, we varied $\sigma$ from 1 to 10 (Supplemental Fig. 7). For the two-opponent model, we generated simulated activations of 150 voxels that have a linear shape; half of them were decreasing (i.e., object-preferring), and another half were increasing across the continuum (i.e., scene-preferring) as a function of depicted spatial scale along the object-to-scene continuum. The intercept of the line was randomly sampled between 0.01 and 0.2, and the slope of the linear line was randomly varied between 0.1 and 1, and half of them were assigned a negative value. These ranges were chosen based on the empirical data; voxel activation was normalized to the grand mean, then for each voxel, we estimated the slope. Among those estimated slopes, we found the minimum and maximum values and normalized them such that the maximum slope becomes 1. For the intercept, we computed the standard deviation of normalized activation and referenced the minimum and maximum values. After creating these hypothetical activations, we extracted distributed patterns of simulated voxels for each condition and computed the pairwise distance (1-Pearson correlation). The PCA and visualization using the RDM and MDS plots were also performed as we did with the actual voxels.

### Comparison between neural and simulation RDMs

To quantify the relationship between the neural RDM and each of model RDM from the simulated data, we measured the correlation between each pair of RDMs. From each RDM, we took the values from the lower triangle and vectorized them. Then, we measured the similarity between each pair using Spearman correlation. This procedure was performed between each subject’s neural RDM and each model RDM. To test which model is more similar to the neural data, we transformed the measured correlation values using Fisher’s Z, then performed a pairwise *t*-test.

## Supplementary Information

Supplementary Information.

## Data Availability

All stimuli are available in an OSF repository (https://osf.io/hcmgk/). The data that support the findings of this study will be publicly available after publication, or upon request to the corresponding author.
