# Supplementary material for: Ramp-shaped neural tuning supports graded population-level representation of the object-to-scene continuum
Source: Sci Rep. 2022 Oct 27;12:18081. doi: 10.1038/s41598-022-21768-2 (PMC9613906; doi:10.1038/s41598-022-21768-2)
Supplement: Supplementary file 1 — Supplementary Information. [file 41598_2022_21768_MOESM1_ESM.pdf]

Supplementary Information

Ramp-shaped neural tuning supports graded population-level representation of the object-to-scene continuum

Jeongho Park[1,*], Emilie Josephs[2], and Talia Konkle[1]

[1]*Department of Psychology, Harvard University*
[2]*Computer Science & Artificial Intelligence Lab, Massachusetts Institute of Technology*
[*]*email: jpark3@g.harvard.edu*

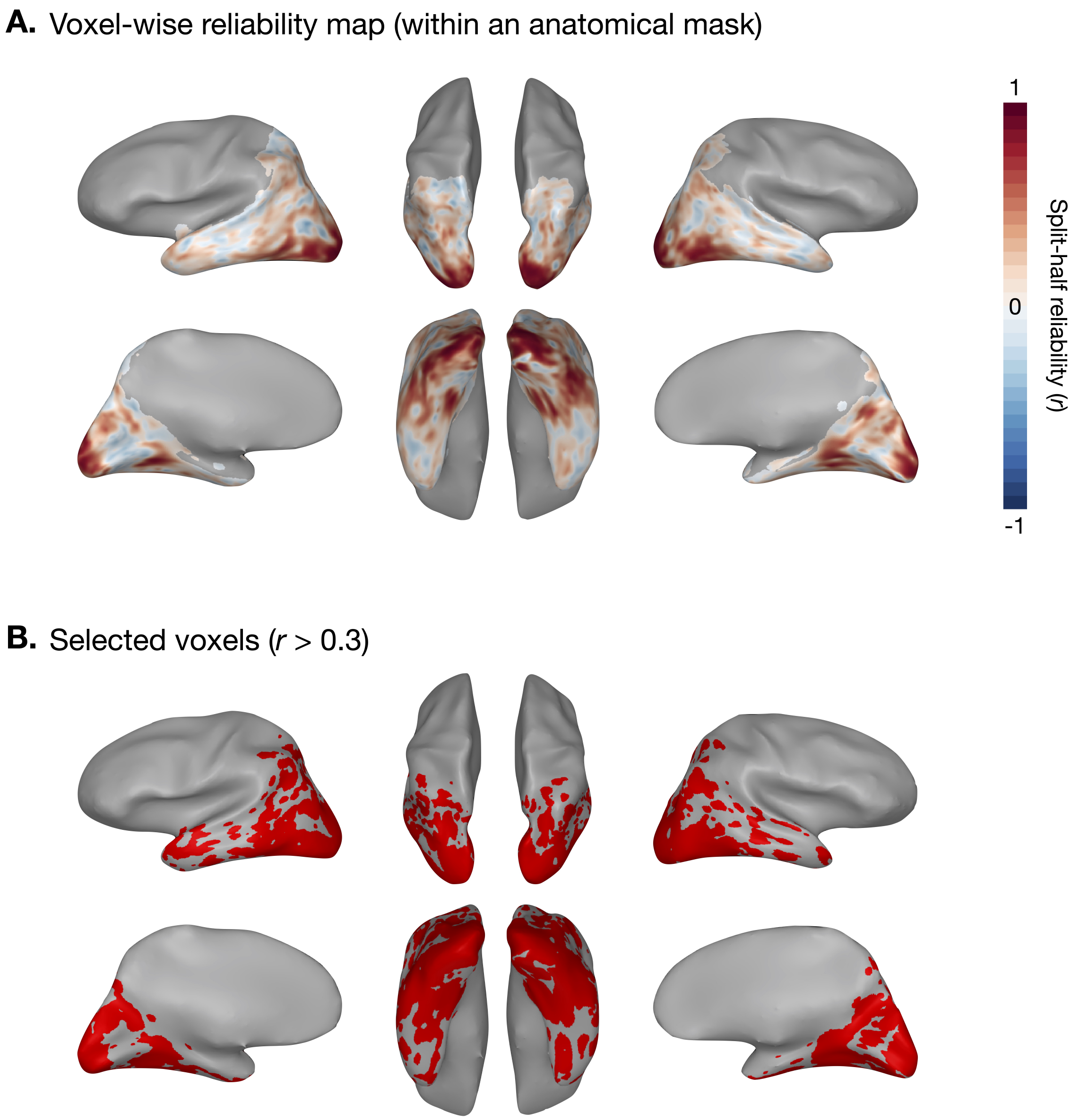

Supplementary Figure 1: *Voxel Selection. (A) First, we manually defined an anatomical mask that includes occipito-temporal cortex, occipito-parietal cortex, and the corresponding medial part of the brain. Within the mask, voxel-wise reliability was measured by correlating the betas between odd and even runs, from a group-level GLM. (B) Voxels that have higher reliability than the threshold (r=0.3) were selected for subsequent analyses. These resulting voxels are shown in red.*

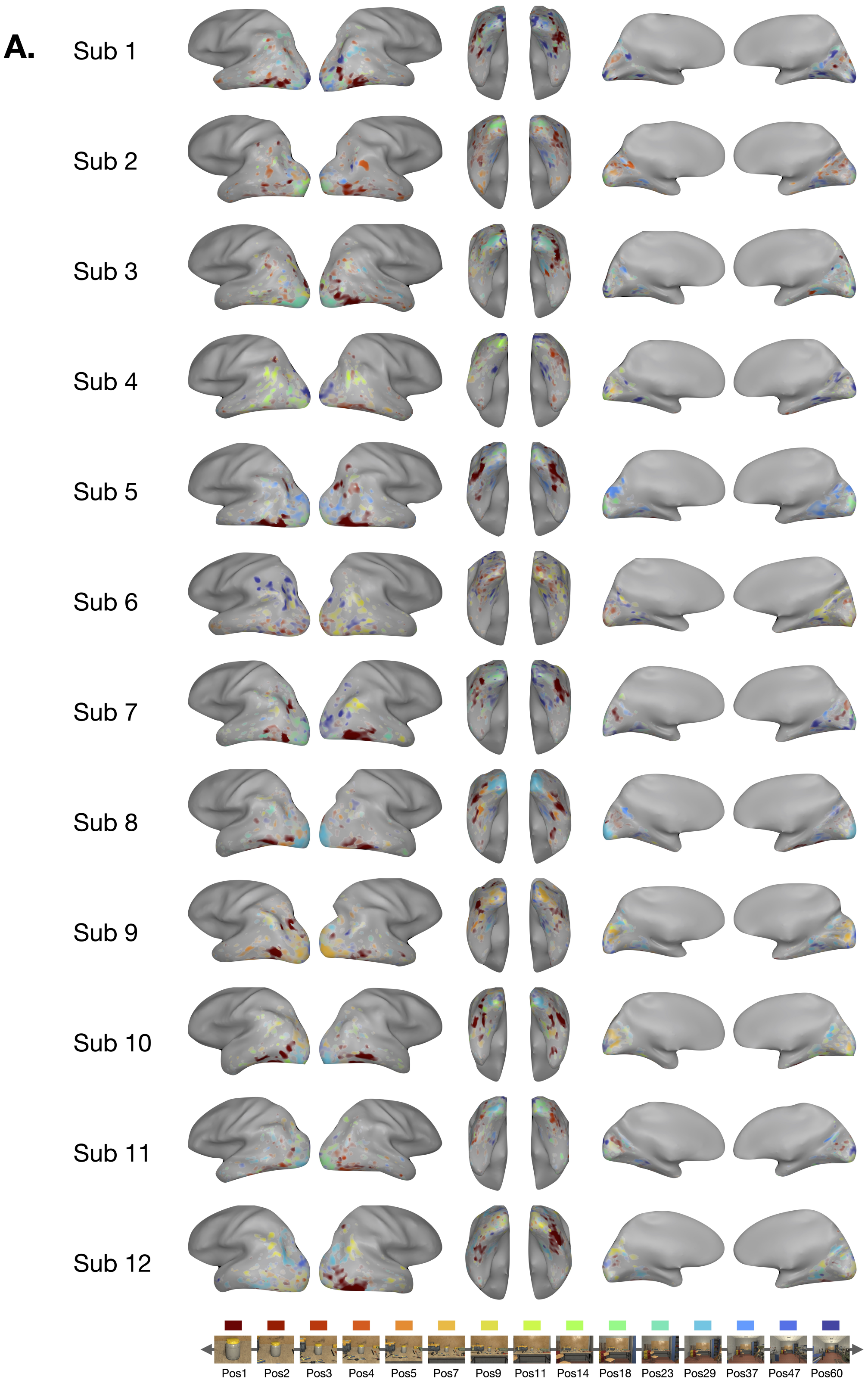
A.
Sub 1
Sub 2
Sub 3
Sub 4
Sub 5
Sub 6
Sub 7
Sub 8
Sub 9
Sub 10
Sub 11
Sub 12
Pos1
Pos2
Pos3
Pos4
Pos5
Pos7
Pos9
Pos11
Pos14
Pos18
Pos23
Pos29
Pos37
Pos47
Pos60

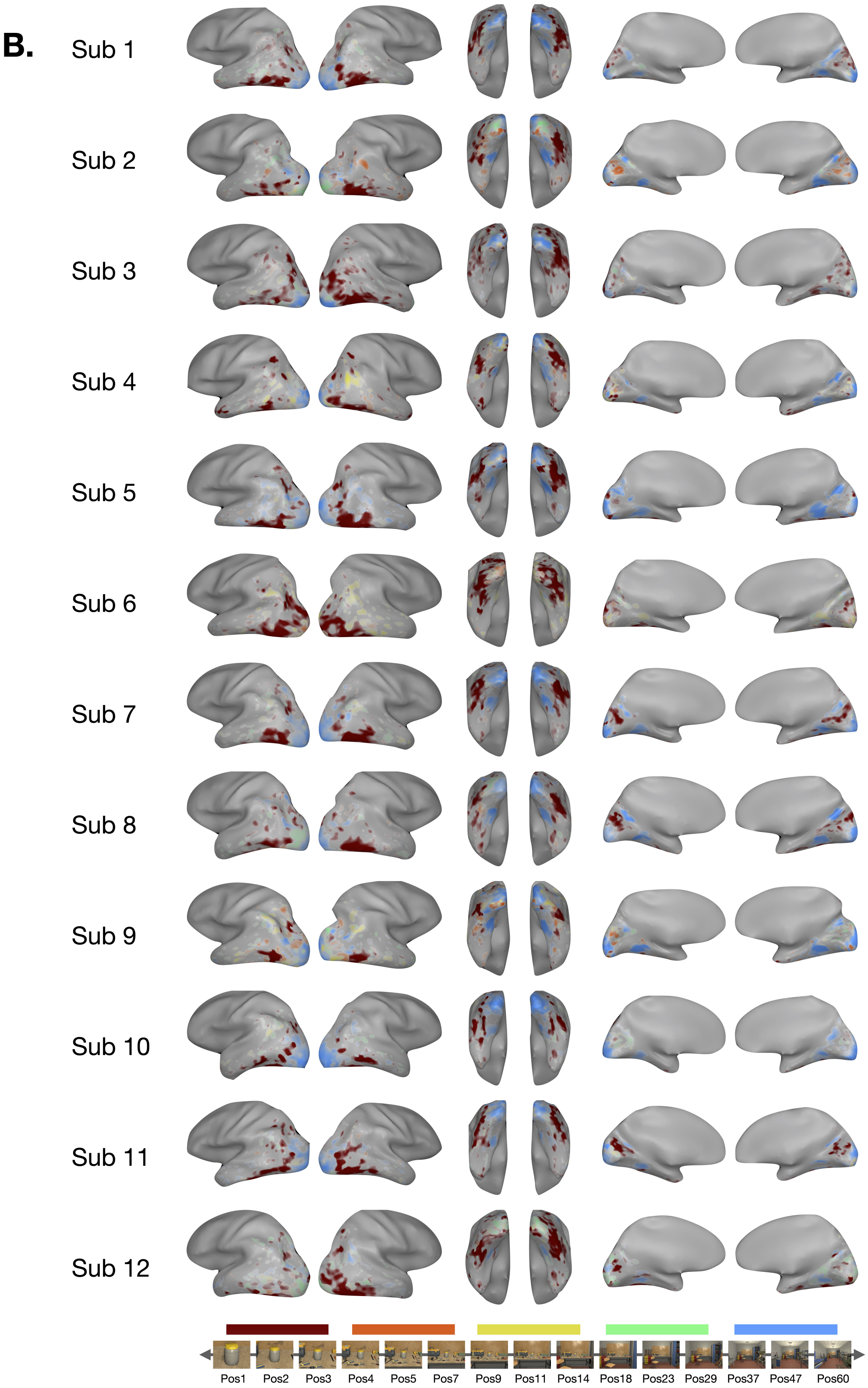

Supplementary Figure 2: *Individual Preference Maps. In addition to the group-level preference map (Figure 2), preference maps were also computed at each individual participant level. (A) Preferences for all conditions (i.e., 15 Positions) were measured, in the same manner as the group data. A lot of variation across participants was observed. (B) Voxels' preferences were grouped and binned for 3 neighboring conditions. For example, if a voxel showed preference for condition 2, it was colored as the same color as voxels that showed preference for condition 1 or condition 3.*

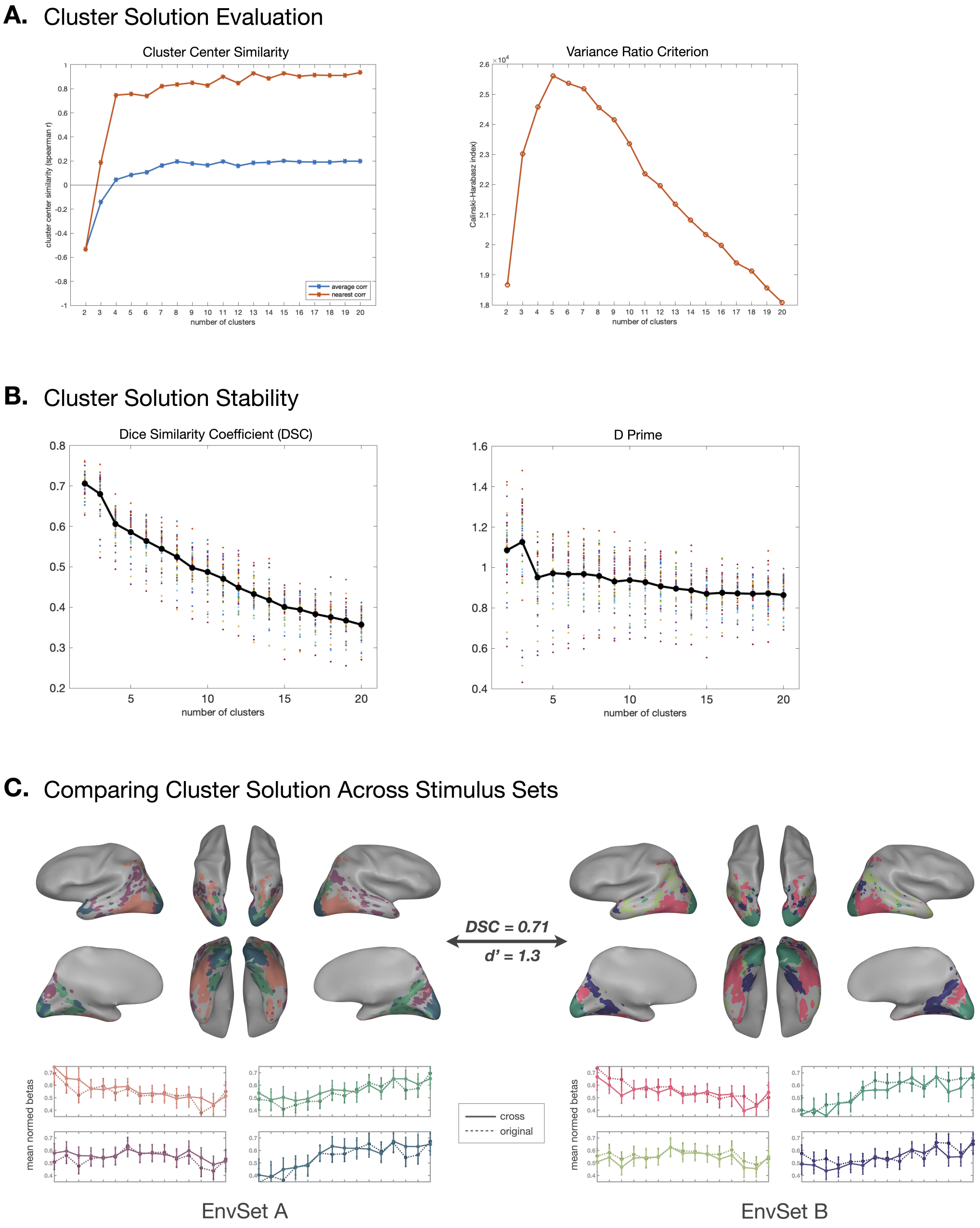

Supplementary Figure 3: *Cluster Solution Evaluation. (A) Left: For each number of clusters, we measured how similar the cluster centers are to one another on average. Right: we also measured the Variance Ratio Criterion (Clainski-Harabasz score), which is a ratio between the within-cluster dispersion and the between-cluster dispersion. (B) The stability of the clustering solutions was assessed across participants. For each k, the participants were randomly split into two groups, and we measured how well one group predicted another, using two different metrics: Dice Similarity Coefficient and D prime. Dice coefficient accounts for both false positives and negatives, whereas the D-prime measures the detection sensitivity against the false positives. This procedure was repeated over 50 iterations, and the mean values were calculated for each k. (C) The reliability of the clustering solution was measured across different subsets of stimuli. The data were split into two based on the stimuli (Environment Set A vs. Environment Set B), and the identical clustering algorithm was run separately for each data set. Then, we compared the voxel cluster assignments between the two solutions by measuring the Dice coefficient and D-prime. Further, we cross-validated the response profile across the two subsets. Underneath the brain map, mean normalized betas from each cluster are shown. The dotted line represents the data from the original subset, where the cluster solution was determined. The solid line represents the data from the other subset, where we extracted betas based on the clustering solution from the original set.*

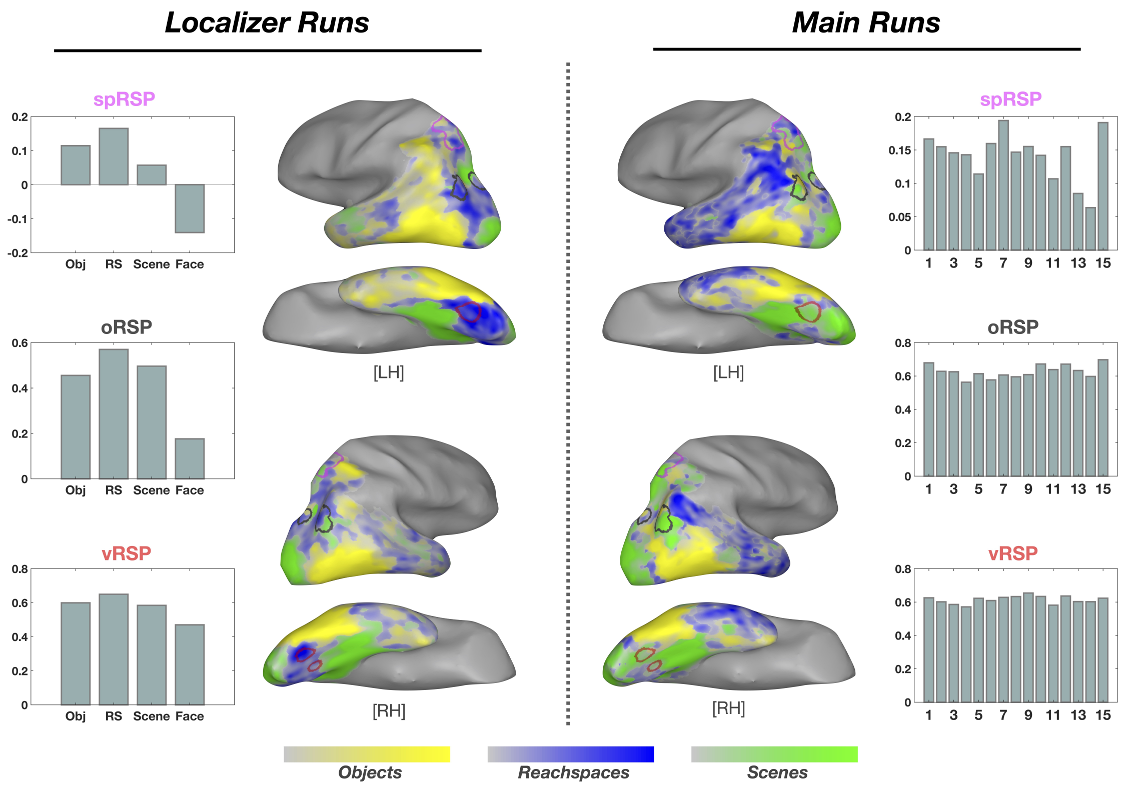

Supplementary Figure 4: *Reachspace-preferring areas. In the current study, we found some mixed evidence regarding previously reported reachspace-preferring areas (Josephs and Konkle, 2020). To directly compare our data to the previous study, we obtained three ROI masks ("protoROIs") from the Josephs and Konkle (2020), in the superior parietal cortex (spRSP, pink), the occipiotal cortex (oRSP, black), and the ventral visual cortex (vRSP, red). First, we examined how these protoROIs responded to our stimulus conditions, separately for localizer runs and main runs. In the localizer, all 3 protoROIs showed the highest activation to the reachspaces condition compared to objects, scenes, or faces (bar graphs, left panel). In the main runs, a similar trend was shown at the superior parietal region (spRSP), but not at the occipito-parietal region (opRSP) or the ventral region (vRSP; bar graphs, right channel). Next, we computed 3-way preference maps (objects, reachspaces, and scenes) with the current study data and compared anatomical locations of the resulting reachspace-preferring cortex to the protoROIs. As the main runs did not have explicitly labeled reachspace conditions, we considered conditions 5-7 (i.e., Position 5, 7, and 9) as the reachspaces, condition 1 (Position 1) as the objects, and condition 15 (Position 60) as the scenes. Then we extracted data corresponding to those conditions and performed the preference mapping procedure. This analysis was performed on all voxels within our group-level anatomical mask (Supplementary Figure 1), without the voxel-reliability thresholding step. Considering the maps from the localizer runs, we observed close correspondence to the maps from Josephs and Konkle (2020). Additionally, most of the protoROIs showed good correspondence with the reachspace-preferring cortex. However, the maps from the main runs showed different topographies from both the localizer run and the preference maps in Josephs and Konkle (2020). The discrepancies between the localizer and main runs could exists for 3 reasons: 1) reduced power in the main runs compared to localizer runs (i.e. fewer fMRI blocks were included in the contrast), 2) possible differences in the responses to real vs CGI images, and 3) possible differences in the reliabilities of the voxel responses to the different stimulus sets. Moreover, this suggests that reachspaces may not be simply characterized in terms of viewing distance or the size of scale, and there might be other fundamental components missing from our CGI images. Further investigation is necessary to answer these questions.*

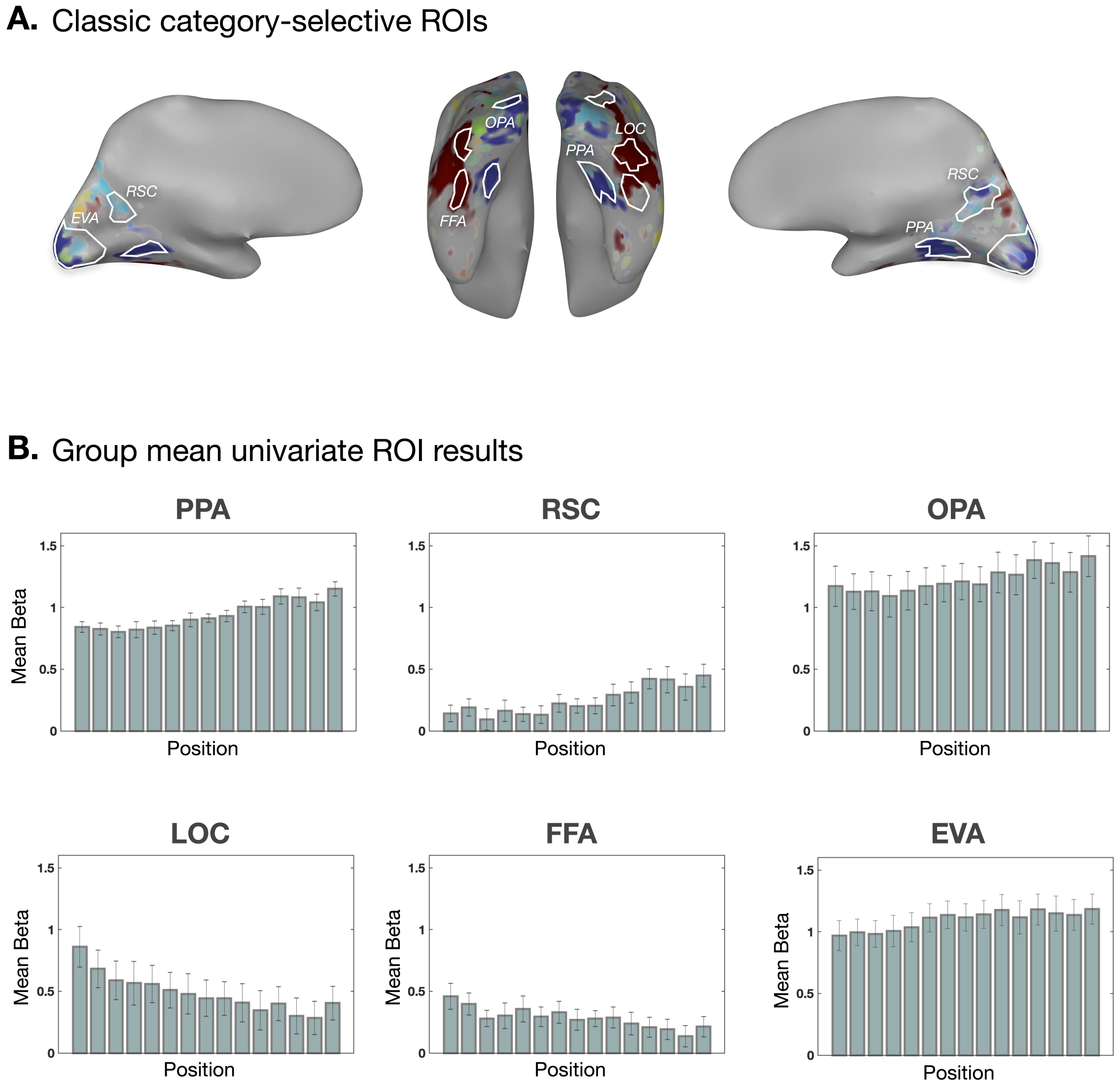

Supplementary Figure 5: *Univariate responses in the ROIs. (A) Anatomical location of classic category-selective ROIs are shown in an exemplary subject's brain. Each ROI was individually defined using the localizer data, and it is marked with a white outline. To provide context to the main results, the ROIs are overlaid on top of the group GLM preference map (shown in Figure 2). (B) Within each ROI, univariate responses were averaged across the voxels for each Position (i.e., depicted spatial scale), then they were averaged across participants. The group mean betas are plotted from object-view to scene-view along the x-axis. The error bar represents standard error across participants. PPA: parahippocampal place area; RSC: retrosplenial cortex; OPA: occipital place area; LOC: lateral occipital complex; FFA: fusiform face area; EVA: early visual areas.*

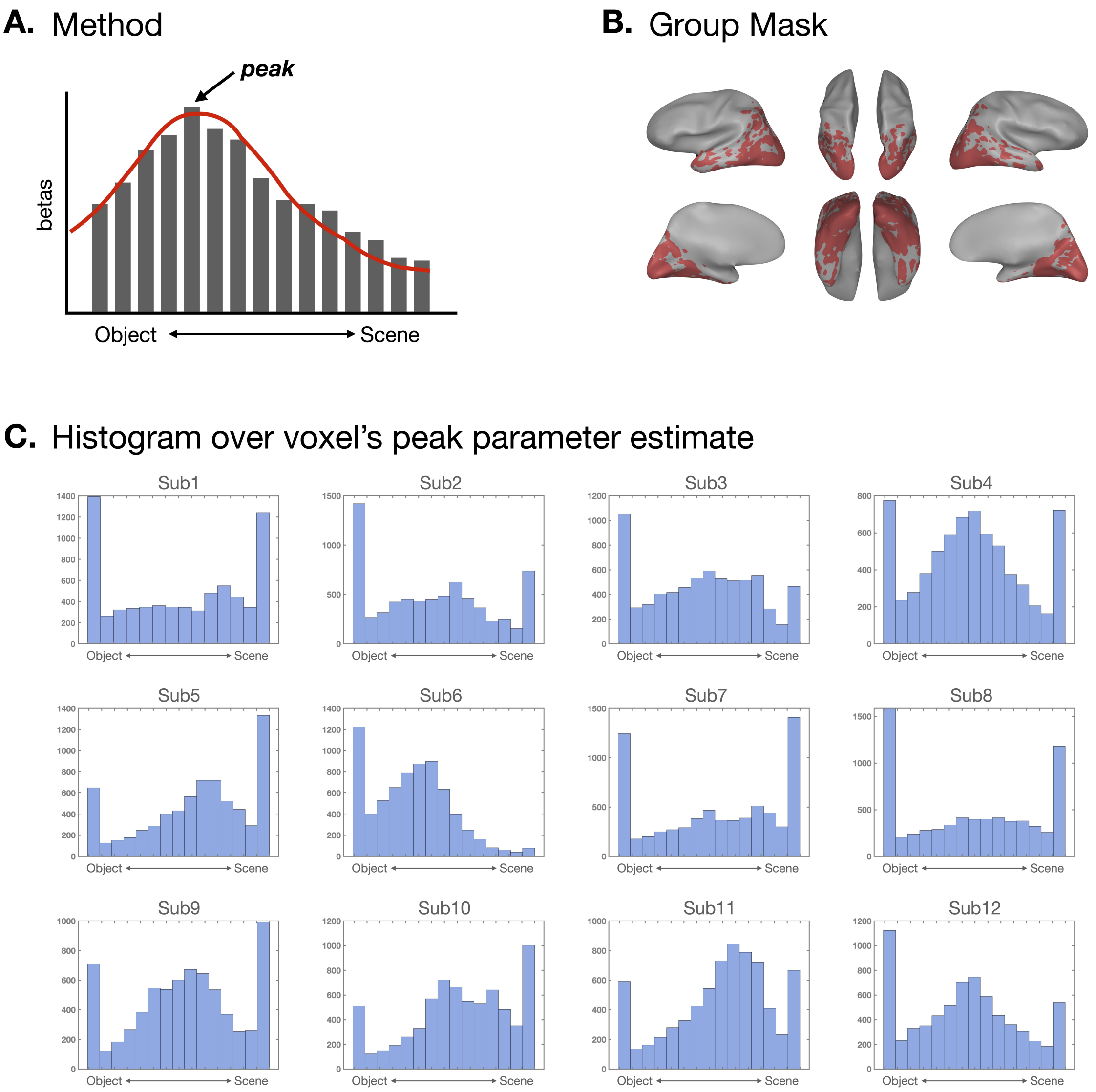

Supplementary Figure 6: *Voxelwise Guassian fit. (A) For each voxel, we estimated its peak by fitting a Gaussian function (bounds of [0 100] for amplitude, [1 15] for center, and [0 15] for width) to the betas across conditions, which were normalized by subtracting its minimum value. (B) Voxels for this analysis were chosen at a group level, based on the split-half reliability (r¿0.3) and an anatomical mask (Fig S1). (C) Histogram of Gaussian fit peaks for each subject. The x-axis represents depicted spatial scale, and the y-axis represents the number of voxels. In most of the subjects, there are two clear peaks at the extreme cases, and some subjects show an additional peak at the intermediate scale. Broadly, these results are consistent with our findings from preference mapping or response profile clustering.*

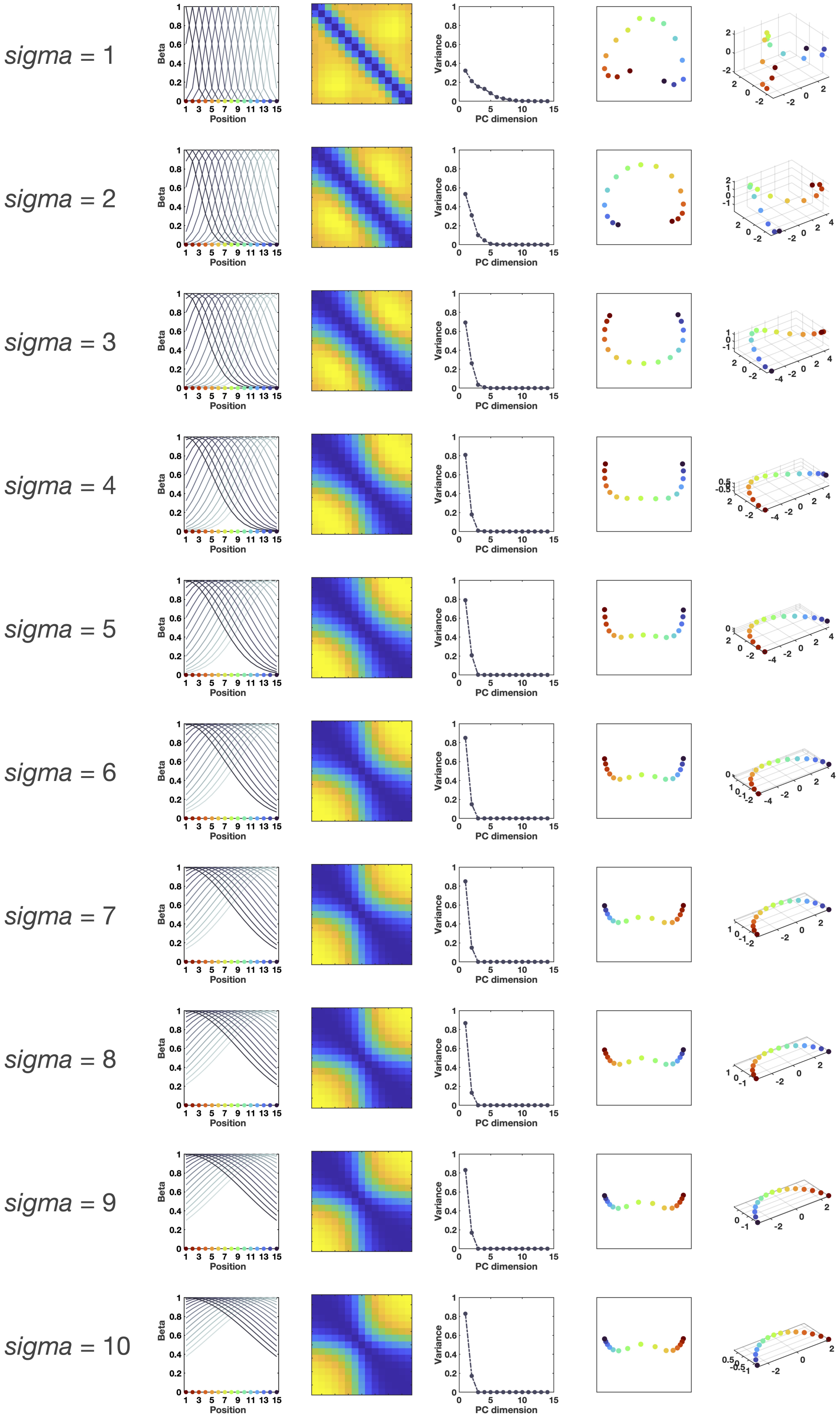

Supplementary Figure 7: *Gaussian tuning simulation with varying width (sigma). We report simulation results when the sigma is systematically varied from 1 to 10. As the sigma gets larger, the representation geometry becomes closer to the ramp-shaped tuning.*

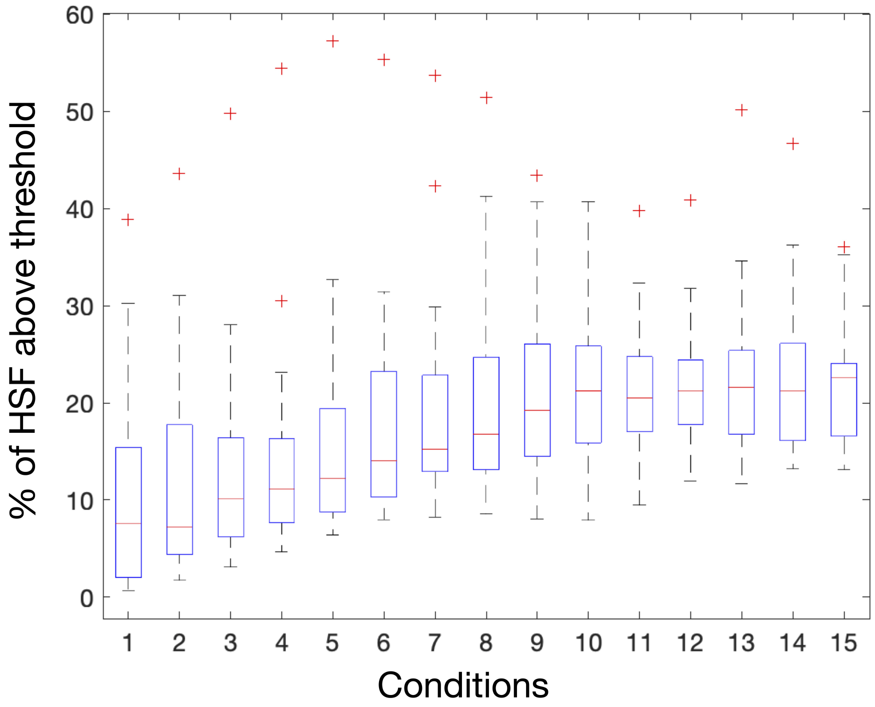

Supplementary Figure 8: *Spatial frequency analysis. The percentage of high spatial frequency above 15 cycle/image was measured from each image, and grouped by the depicted spatial scale.*

k = 2

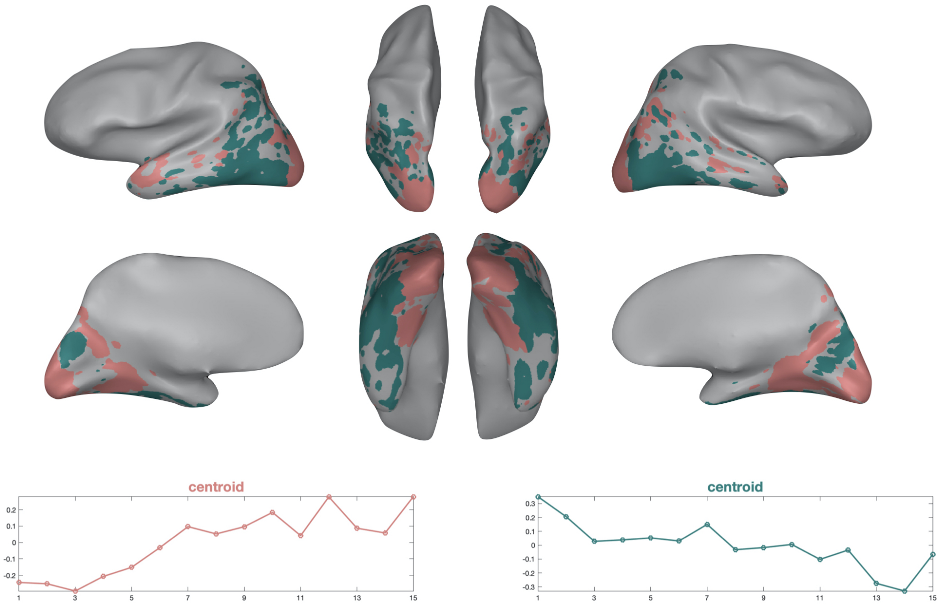

---

k = 3

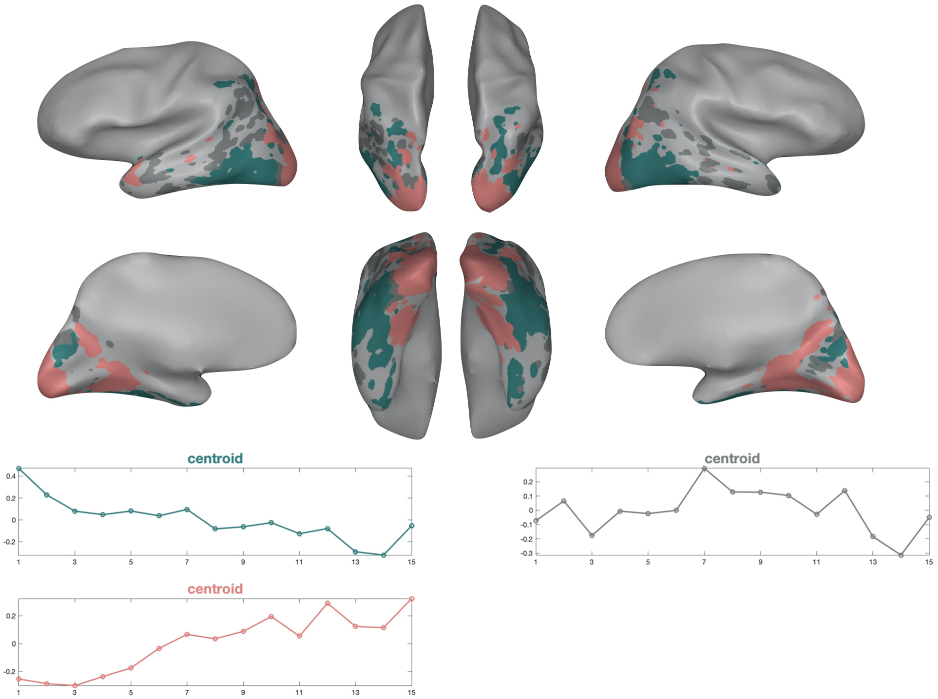

k = 4

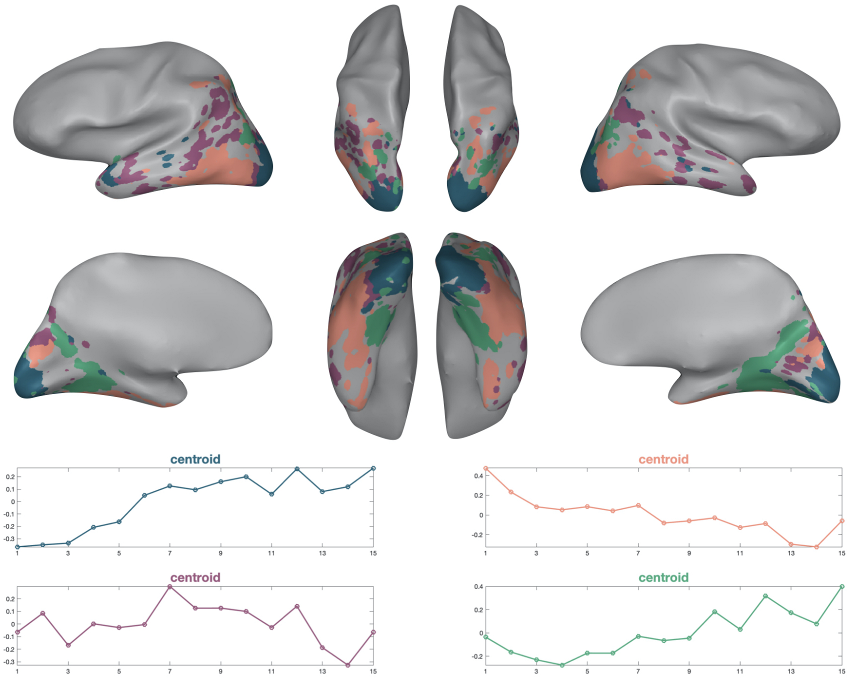
centroid
centroid
centroid
centroid

k = 5

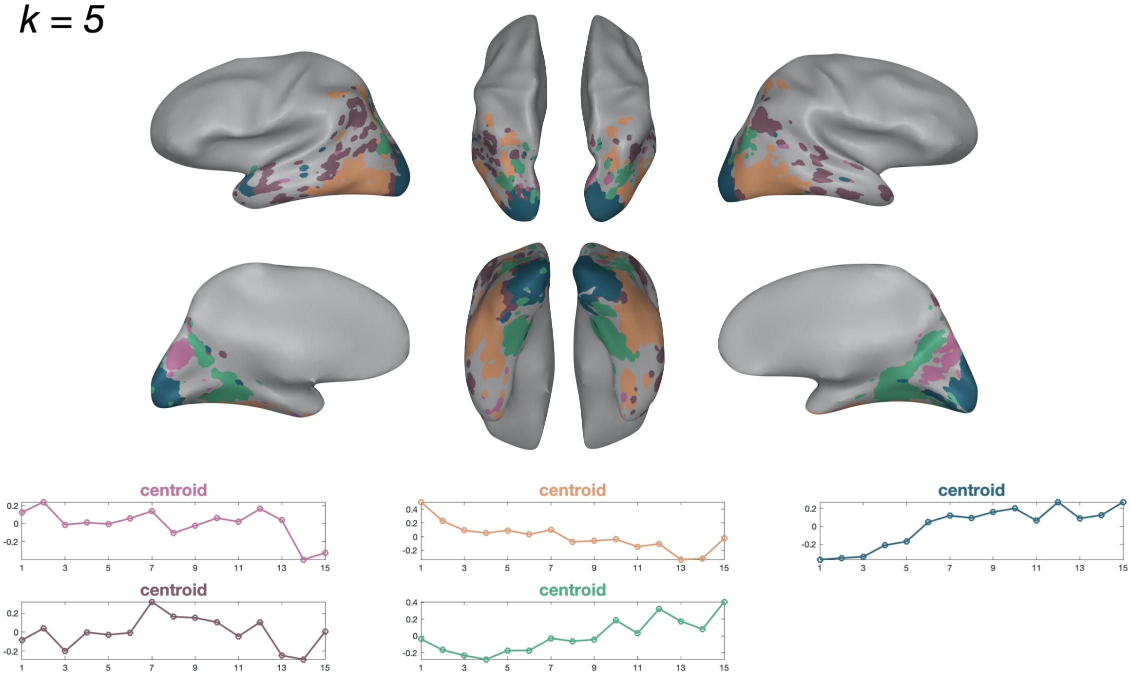
centroid
centroid
centroid
centroid
centroid

k = 6

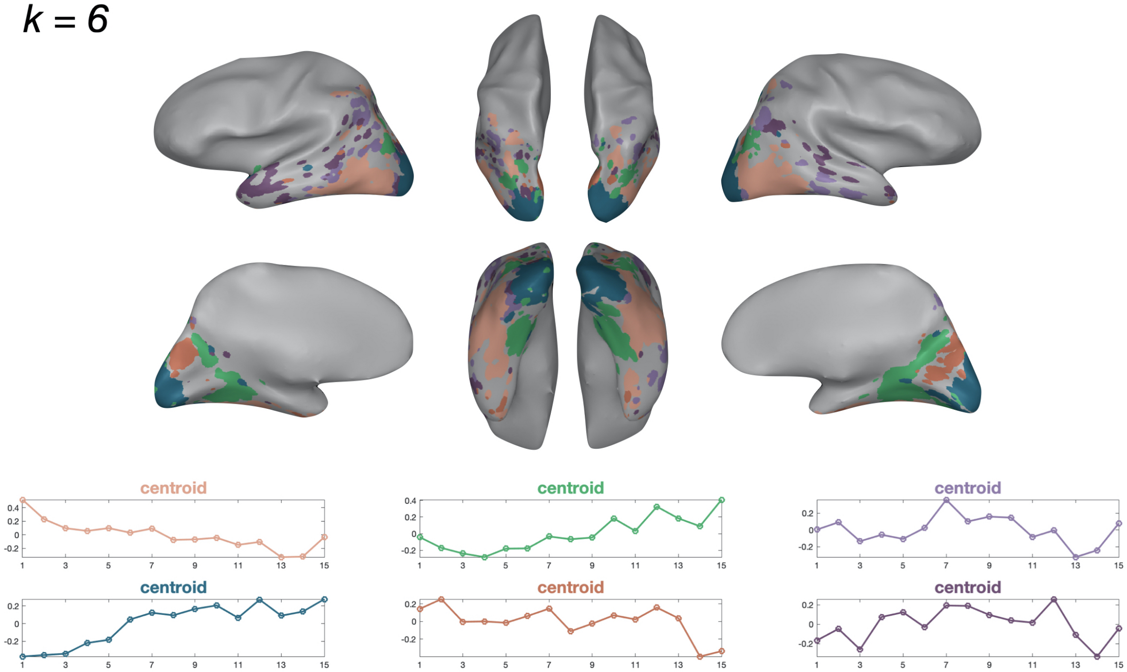
centroid
centroid
centroid
centroid
centroid
centroid

k = 7

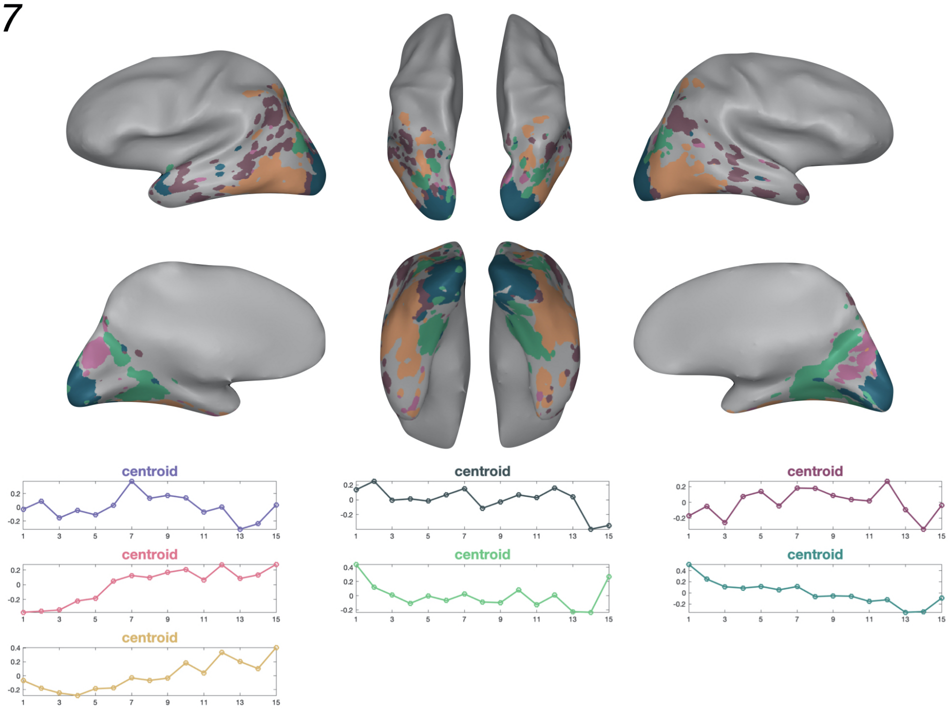
centroid
centroid
centroid
centroid
centroid
centroid
centroid

k = 8

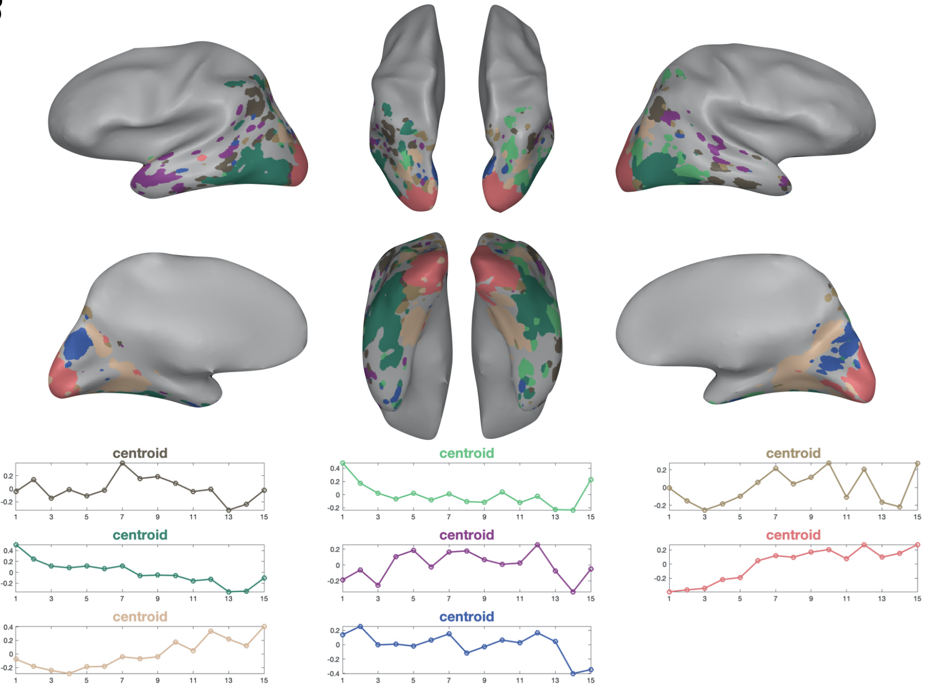
centroid
centroid
centroid
centroid
centroid
centroid
centroid
centroid

k = 9

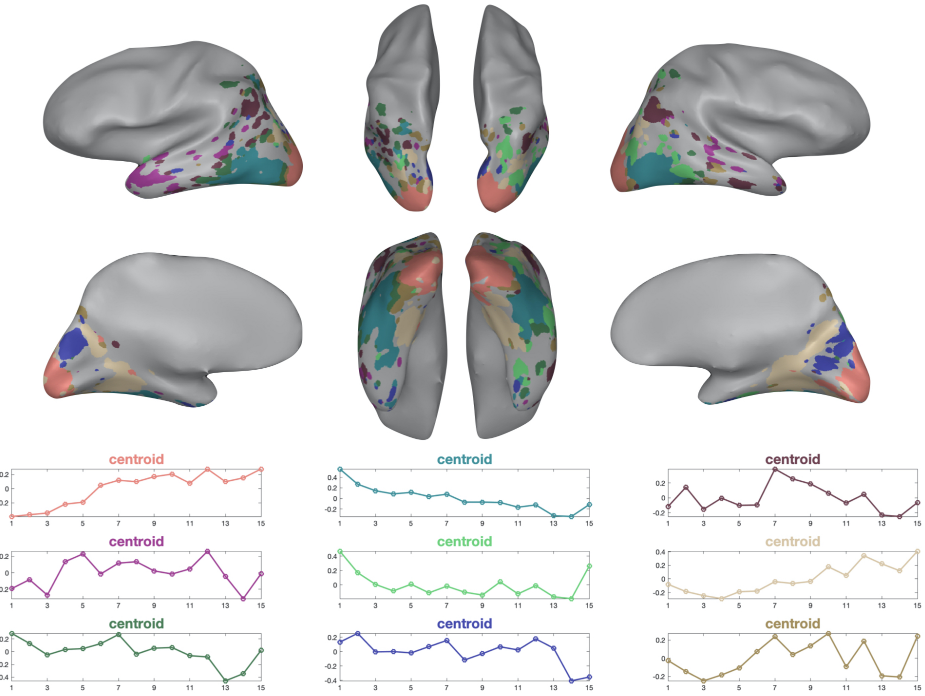
centroid
centroid
centroid
centroid
centroid
centroid
centroid
centroid
centroid

k = 10

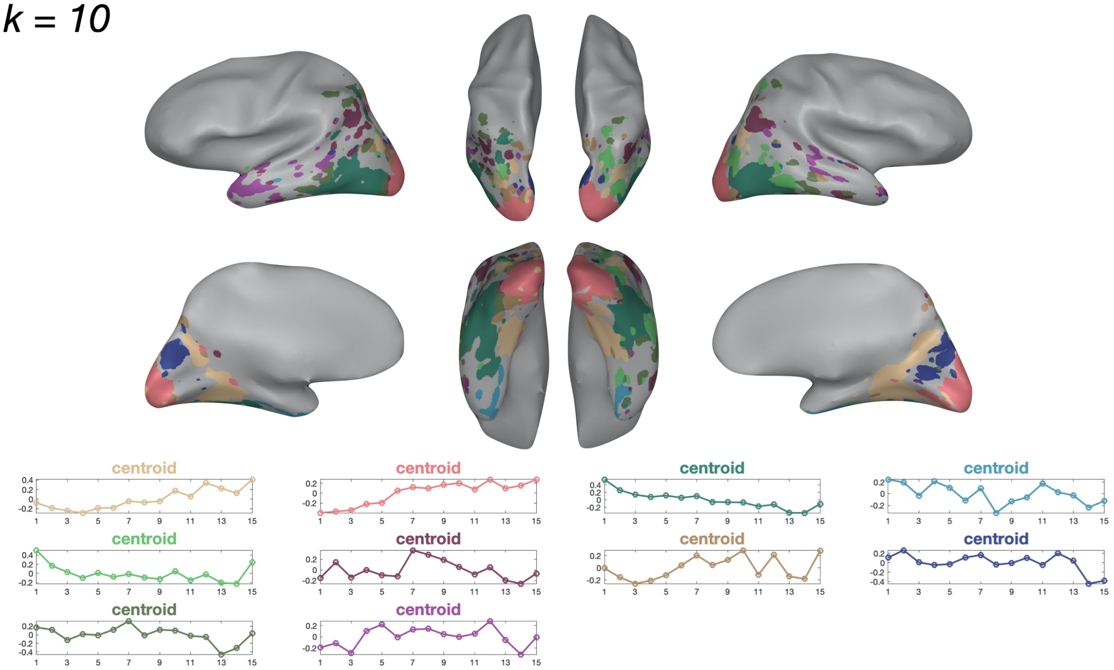
centroid
centroid
centroid
centroid
centroid
centroid
centroid
centroid
centroid
centroid

k = 11

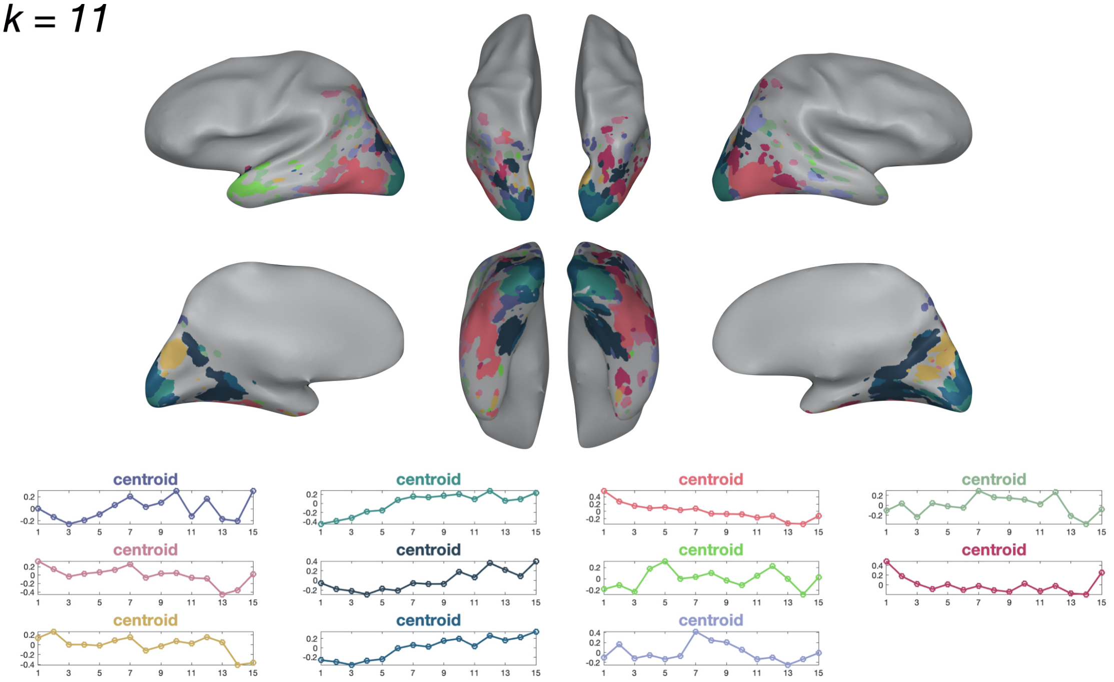
centroid
centroid
centroid
centroid
centroid
centroid
centroid
centroid
centroid
centroid
centroid

k = 12

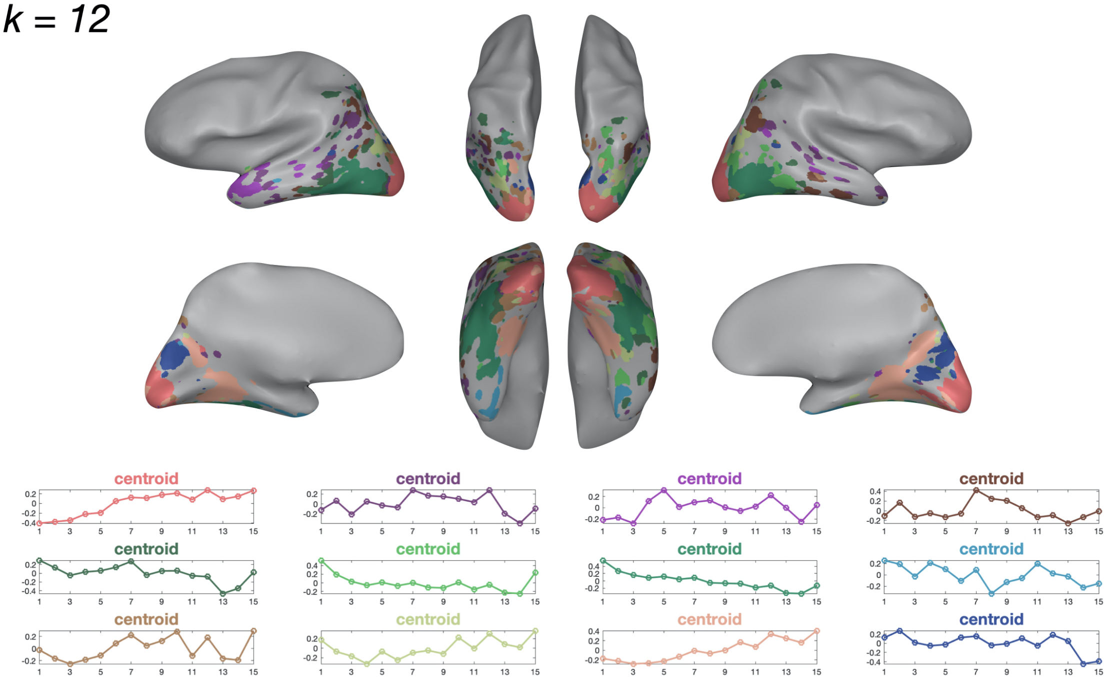
centroid
centroid
centroid
centroid
centroid
centroid
centroid
centroid
centroid
centroid
centroid
centroid

k = 13

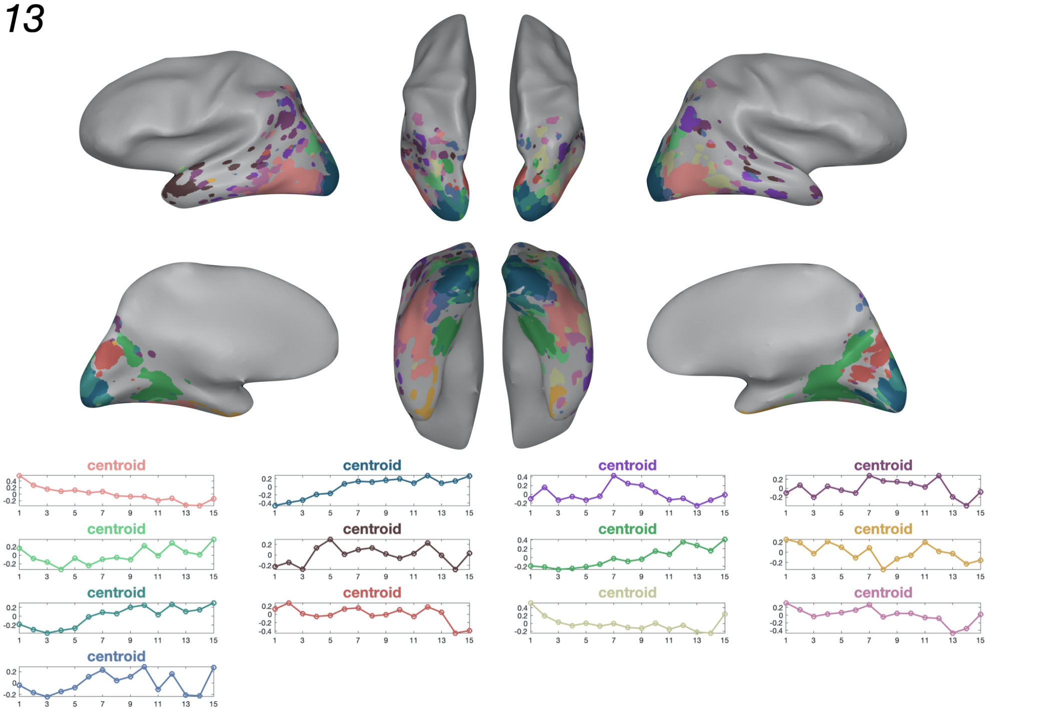
centroid
centroid
centroid
centroid
centroid
centroid
centroid
centroid
centroid
centroid
centroid
centroid
centroid

k = 14

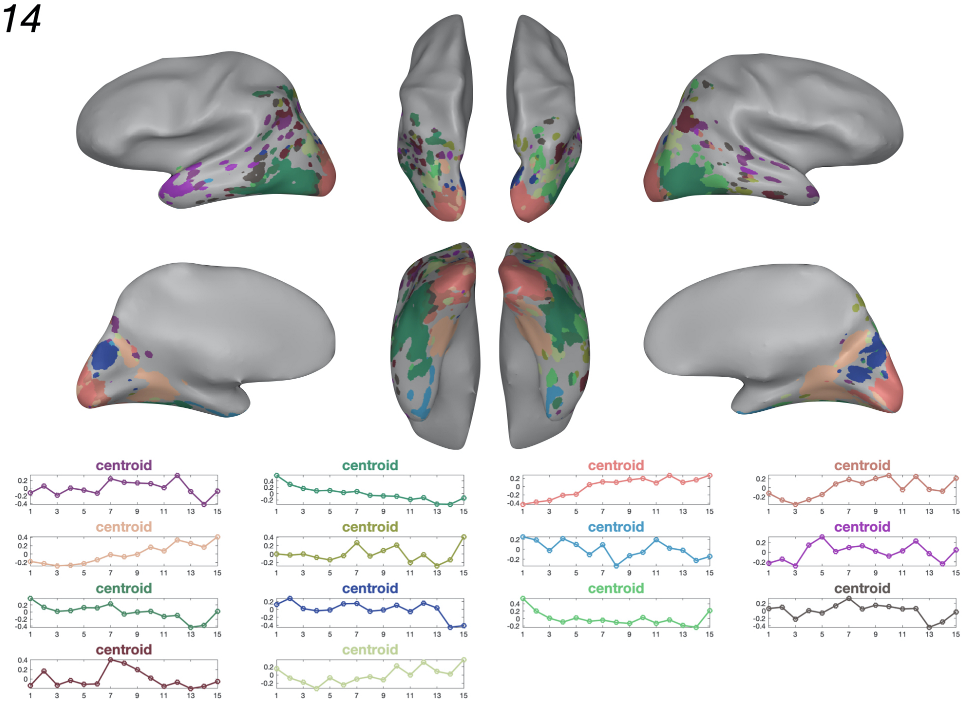
centroid
centroid
centroid
centroid
centroid
centroid
centroid
centroid
centroid
centroid
centroid
centroid
centroid
centroid

k = 15

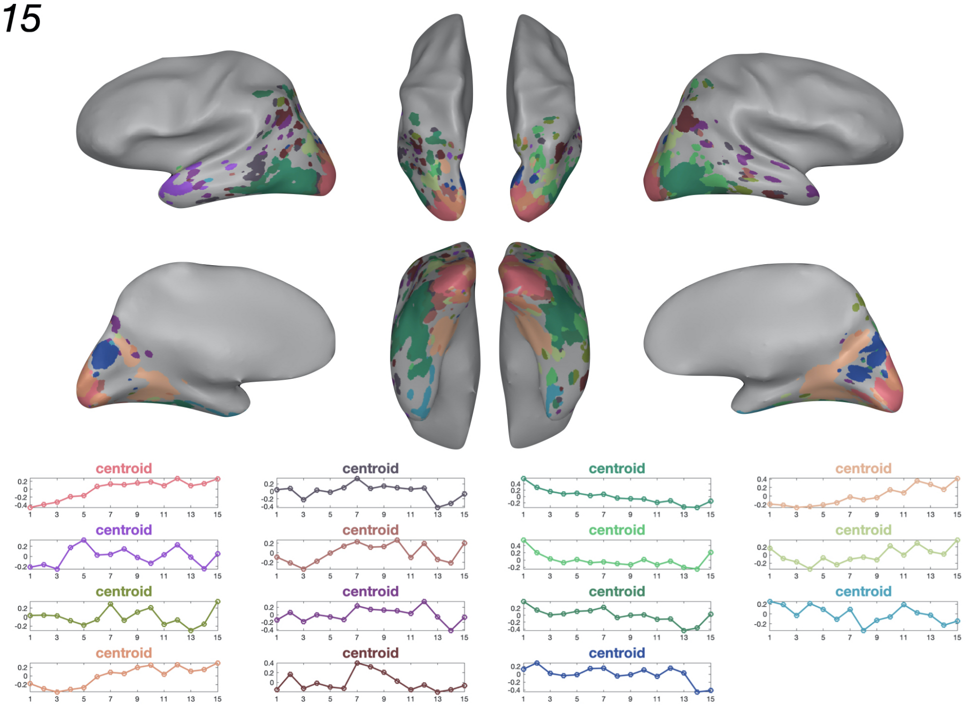
centroid
centroid
centroid
centroid
centroid
centroid
centroid
centroid
centroid
centroid
centroid
centroid
centroid
centroid
centroid

k = 16

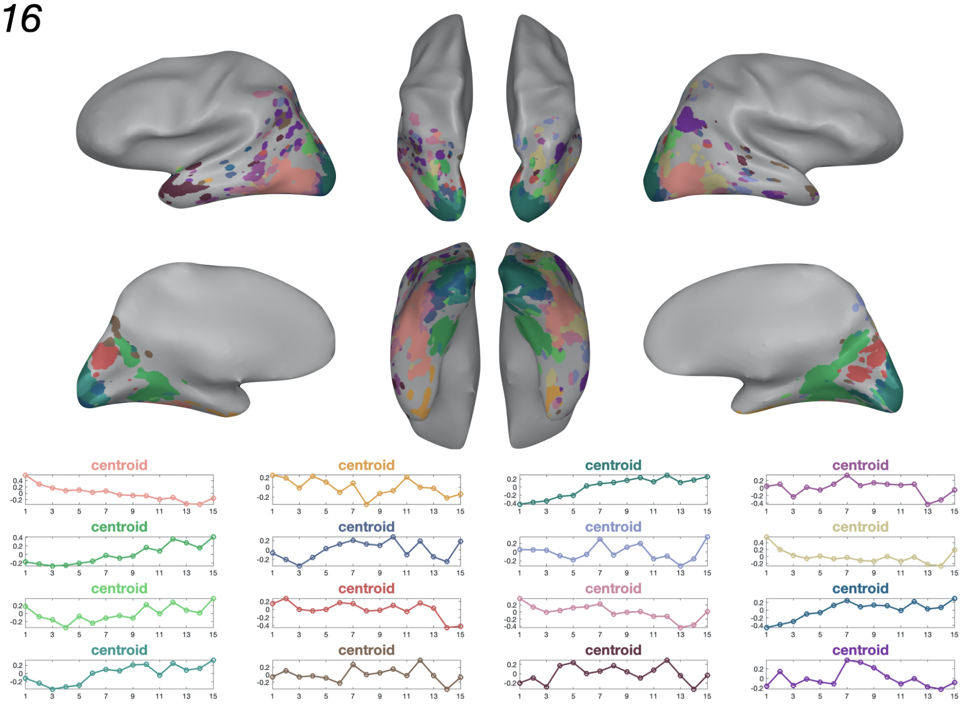
centroid
centroid
centroid
centroid
centroid
centroid
centroid
centroid
centroid
centroid
centroid
centroid
centroid
centroid
centroid
centroid

k = 17

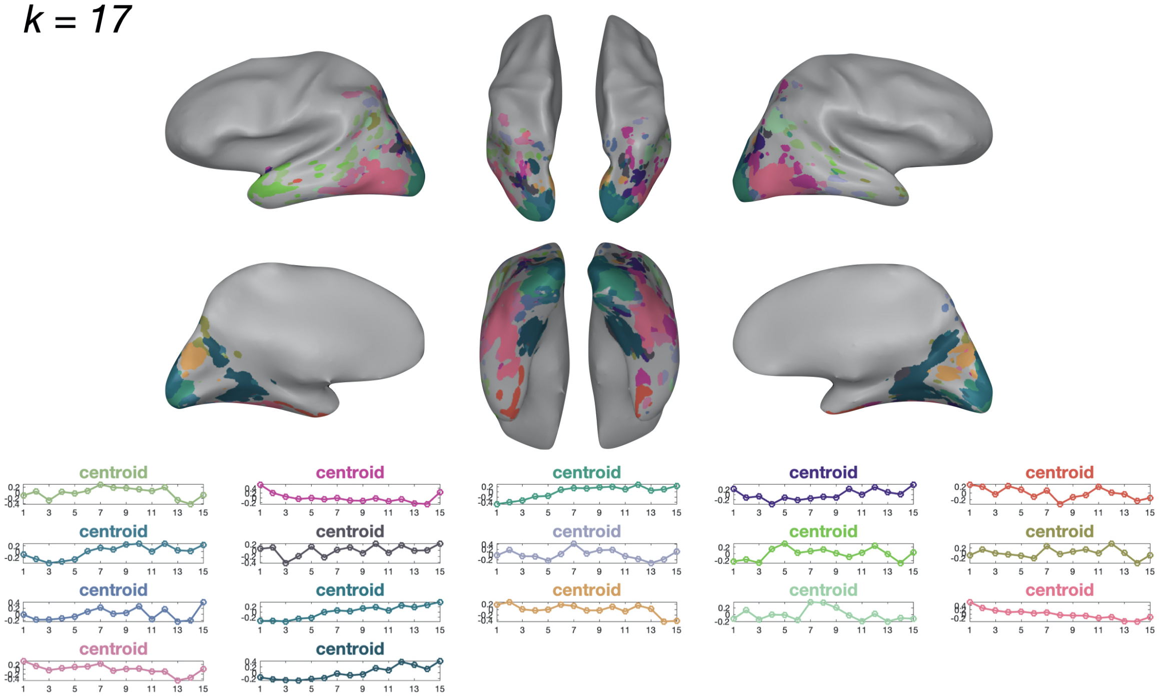
centroid
centroid
centroid
centroid
centroid
centroid
centroid
centroid
centroid
centroid
centroid
centroid
centroid
centroid
centroid
centroid
centroid

k = 18

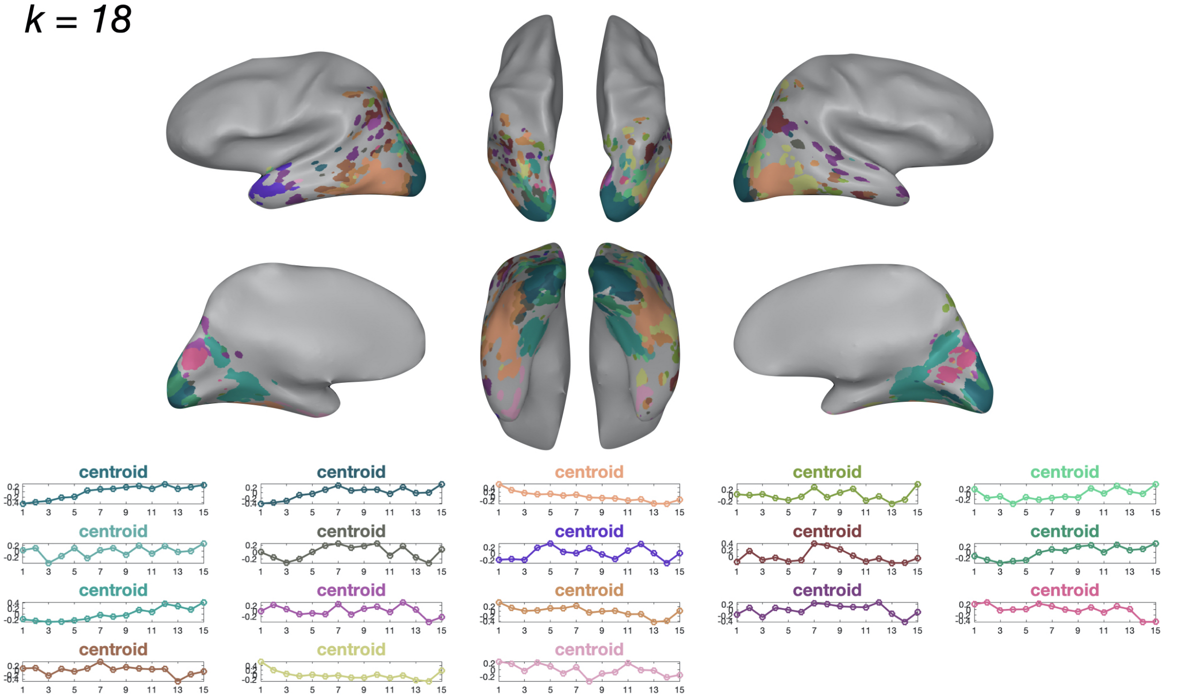
centroid
centroid
centroid
centroid
centroid
centroid
centroid
centroid
centroid
centroid
centroid
centroid
centroid
centroid
centroid
centroid
centroid
centroid

k = 19

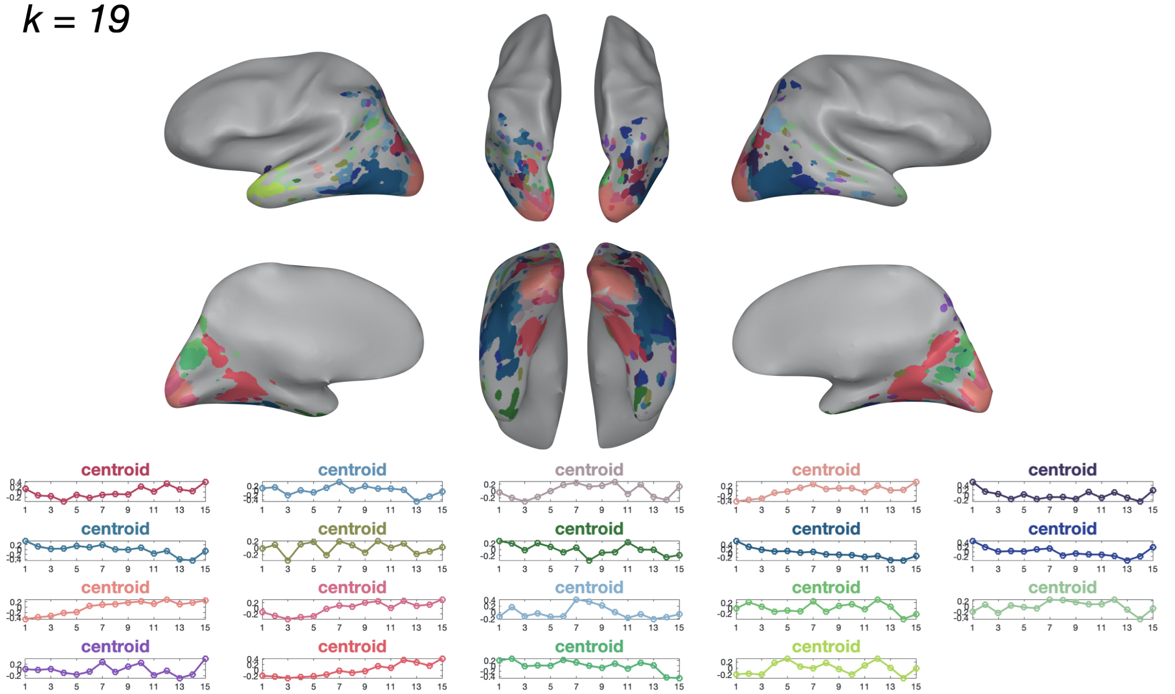
centroid
centroid
centroid
centroid
centroid
centroid
centroid
centroid
centroid
centroid
centroid
centroid
centroid
centroid
centroid
centroid
centroid
centroid
centroid

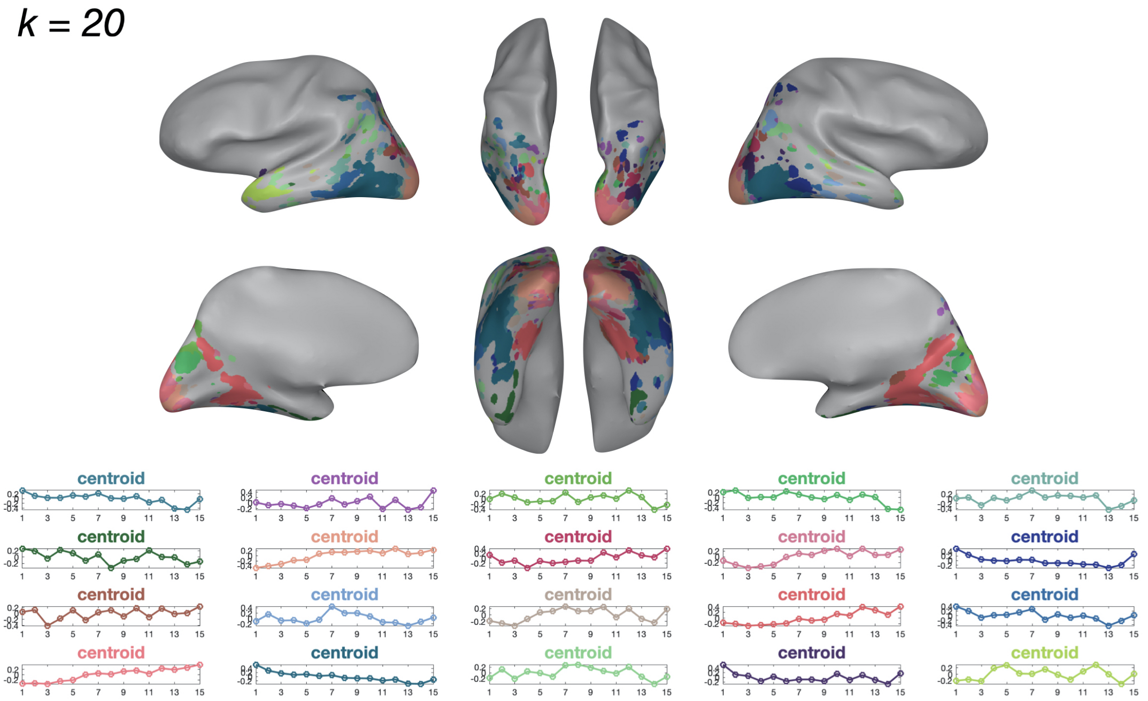

Supplementary Figure 9: *Response Profile Clustering Solutions. Clustering results for all solutions, from k=2 to k=20. Voxels assigned to the same cluster were colored the same, and we chose the colors such that clusters with more similar response profiles were more similar in hue. The corresponding cluster centroids are plotted underneath the cortical map.*
